# A global review of Livestock Guardian Donkeys (LGDks) in the prevention of carnivore depredation

**DOI:** 10.7717/peerj.21265

**Published:** 2026-06-24

**Authors:** Alessandra J. Metzler, Laurie Marker, Robin M. Cook, Bogdan Cristescu

**Affiliations:** 1 University of New England, Armidale, New South Wales, Australia; 2 Cheetah Conservation Fund (CCF), Otjiwarongo, Otjozondjupa, Namibia; 3 School of Animal, Plant and Environmental Sciences, University of Witwatersrand, Johannesburg, South Africa; 4 School of Agriculture and Natural Resources Sciences, Namibia University of Science and Technology, Windhoek, Namibia; 5 School of Applied Sciences, University of Brighton, Brighton, United Kingdom

**Keywords:** Carnivore conservation, Conflict mitigation, Depredation mitigation, Human-wildlife conflict, Livestock depredation, Livestock guardians, Livestock guardian donkey, Non-lethal methods

## Abstract

Conflict between humans and carnivores, known as Human-Carnivore Conflict (HCC), has existed throughout history, with recent escalations attributed to anthropogenic expansion and intensified resource competition. Conflict with carnivores primarily stems from livestock depredation, resulting in economic losses that threaten livelihoods. These conflicts pose significant challenges for affected human communities and carnivore conservation. Non-lethal mitigation strategies, particularly livestock guardian animals (LGAs), are widely used, yet rigorous tests of species-specific effectiveness remain limited. Among LGAs, livestock-guardian donkeys (LGDks) are especially understudied despite widespread use. This paper synthesises findings from an extensive review of 58 global studies on LGDk use and efficacy, examining contextual factors influencing their success and the carnivore species involved in depredation events. We found substantial research gaps, with only 5.1% of reviewed literature consisting of experimental studies. Research discrepancies were also identified across carnivore species involved in depredation events, which encompassed six carnivore families and 27 wild species, including seven classified as threatened. Our findings indicate that donkeys can be effective livestock guardians due to their protective herding instincts, accessibility, cost-effectiveness, and low maintenance requirements. However, critical knowledge gaps persist regarding their practical application, access to reliable information, and tested use in diverse contexts, which could either impede or enhance their effectiveness. Empirical research is needed to refine effective non-lethal strategies, enhance human-carnivore coexistence, and better inform carnivore conservation efforts.

## Introduction

Conflict between humans and predators has existed throughout history due to competition and predation; we refer to this broadly as Human-Carnivore Conflict (HCC), noting that in some systems raptors also take livestock (Eklund et al., 2017; Treves & Karanth, 2003). HCC incidents have increased in recent decades with anthropogenic expansion and intensified resource competition, creating complex, widespread challenges for pastoral communities and for carnivore conservation (Ripple et al., 2014; Ullah et al., 2024; Van Eeden et al., 2018). These trends underscore the need for effective, non-lethal strategies to reduce livestock losses. HCC often results in persecution of large carnivores, driven by both perceived and actual risks to people and their livelihoods, including attacks on people, predation of livestock and pets, direct economic losses, opportunity costs (*e.g.*, time spent guarding, restricted grazing or land use), and intangible costs, including emotional and psychological strain associated with living alongside carnivores (Di Minin et al., 2016; Eklund et al., 2017; Van Eeden et al., 2018). In many cases, predation-related losses disproportionately impact remote, rural communities that are least able to bear the costs (Braczkowski et al., 2023; Di Minin et al., 2016; Drouilly, Nattrass & O’Riain, 2023; McManus et al., 2015; Rigg, 2001). To mitigate losses, local communities commonly use readily available lethal, and often ineffective, means either proactively or in retaliation (Eklund et al., 2017; Van Eeden et al., 2018; Drouilly, Nattrass & O’Riain, 2023). Consequently, the widespread persecution of carnivore species (herein ‘carnivores’) has profound implications for their conservation (Di Minin et al., 2016; Ripple et al., 2014; Ugarte, Moreira-Arce & Simonetti, 2019). As a globally threatened taxon, species within the order *Carnivora* face increased risks, compounded by the loss or reduction of their crucial roles in shaping ecosystem function and health (Di Minin et al., 2016; Inskip & Zimmerman, 2009; Janeiro-Otero et al., 2020; Lazure & Weladji, 2024).

In response to increasing carnivore persecution and conflict, there is growing recognition that non-lethal mitigation strategies are essential for human communities and carnivores to coexist sustainably (Eklund et al., 2017; Naha et al., 2020; Ullah et al., 2024; Van Bommel & Johnson, 2023). Non-lethal methods, especially livestock guardian animals (LGAs) such as dogs, llamas/alpacas, and donkeys, are widely used to reduce livestock predation by staying with herds, detecting intruders, and deterring approach (Andelt, 2004; Smith et al., 2000; Van Bommel & Johnson, 2023). Evidence for LGAs is promising but mixed, and many non-lethal practices still lack rigorous tests (Breitenmoser et al., 2005; Eklund et al., 2017; Treves, Krofel & McManus, 2016; Van Bommel & Johnson, 2023).

Within LGAs, livestock-guardian donkeys (LGDks) (*Equus asinus*) are gaining attention for traits relevant to canid deterrence, vigilance, a low flight response, and dog-averse behaviours, and for potential cost advantages relative to guard dogs in some systems (Andelt, 2004; Smith et al., 2000; Walton & Feild, 1989). However, most reports on LGDks come from surveys and producer accounts, and systematic, empirical evaluations remain limited (Botha, 2018; Green, 1989; Luyt, 2020; Marker-Kraus et al., 1996; Walton & Feild, 1989; O’Brien, Lansdowne & Cook, 2014). These gaps hinder best-practice guidance and underscore the need for targeted research and evaluation.

### Donkeys as livestock guardians (LGDks)

Reports of donkeys deterring canids on rangelands appear widely from the late 1970s–1980s in North America, where extension surveys described variable but sometimes substantial reductions in livestock predation by coyotes (*Canis latrans*) and free-ranging, feral or domestic dogs (herein ‘dogs’) (*Canis familiaris*), especially in small, open pastures with a single jenny or gelding bonded to stock (Andelt, 2004; Smith et al., 2000; Walton & Feild, 1989). Similar use has been documented in Australia against dingoes (*Canis lupus dingo*), and dogs, with outcomes contingent on individual temperament, bonding, and paddock size (Bough, 2016; Jenkins, 2003; P. Gibbs, pers. comm., 2024). In southern Africa, producer accounts and program reports note reductions in black-backed jackal (*Lupulella mesomelas*) (herein ‘jackal’) and caracal (*Caracal caracal*) losses following adoption of donkeys, sometimes alongside dogs (Botha, 2018; Luyt, 2020; Marker-Kraus et al., 1996). Mechanisms proposed include vigilance, braying/alarm, approach and pursuit, and a low flight response to canids; however, the evidence base remains limited, with few controlled evaluations relative to guard dogs and other LGAs (Eklund et al., 2017; Treves, Krofel & McManus, 2016; Van Bommel & Johnson, 2023).

Donkeys are valued as livestock guardians for vigilance, a low-flight/“stand and face” response to canids, and deterrent behaviours (alarm braying, chasing, striking) that can disrupt predation attempts, particularly by coyotes and free-ranging dogs (Bourne, 1994; Gese, Hart & Terletzy, 2021; Green, 1989; Landry, 2000; Marker-Kraus et al., 1996; Ontario Ministry of Agriculture, Food and Rural Affairs (OMAFRA), 2020; Scasta et al., 2024). They also offer practical advantages in the forms of hardiness in harsh climates, diets aligned with small-stock systems, long working lives, and lower training/maintenance costs than some alternatives (Braithwait, 1996; Coppinger & Coppinger, 2014; Jenkins, 2003; Van Bommel & Johnson, 2023). Donkeys are social, form strong bonds (*e.g.*, jenny-foal), and readily affiliate with livestock; these social tendencies underpin their guarding role (Dohner, 2007; Asa, Marshall & Fischer, 2012; Bough, 2011; Burden & Thiemann, 2015; Murray, Byrne & D’Eath, 2013). Territorial guarding instincts can also create conflicts with unfamiliar stock or farm dogs if unmanaged (Bough, 2016; Landry, 2000; Lõoke et al., 2024). Selection and bonding are therefore critical: producers typically choose jennies or geldings and implement a short, structured bonding period (around 6 weeks) kraaling the donkey with its herd, minimising handling, supervising initial dog exposures, and then gradually expanding range once the animal reliably stays with and does not harass stock (Bourne, 1994; Braithwait, 1996; Green, 1989; P Gibbs, pers. comms., 2024). These strengths, and the risks when selection and bonding are poor, indicate clear potential but context-dependent performance, and the evidence remains fragmented and largely non-experimental. This context motivates the present review.

This review addresses critical gaps by (i) synthesising evidence on LGDk deployment and outcomes, (ii) assessing their effectiveness and constraints as a non-lethal predation-reduction tool, and (iii) outlining practical guidance and research priorities. Although donkeys are widely used in conflict mitigation, particularly in resource-limited settings, empirical and species-specific evidence of their effectiveness remains scarce. As global conservation efforts increasingly prioritise coexistence with carnivores, synthesising current knowledge on LGDks is essential. We analyse a comprehensive dataset of literature to examine LGDk use and reported efficacy around the globe in different ecological contexts. These contexts include geographic regions, methodologies for assessing efficacy, associated carnivore species, livestock variables, and the reported success or limitations of LGDk use in reducing predation. This review is intended for both academic researchers and non-academic stakeholders, including conservationists, policymakers, and livestock owners, to provide insight and guidance on strategies that support both human livelihoods and carnivore conservation using LGDks.

## Methods

### Literature selection process

In conducting this literature review, we applied strict eligibility criteria: we included records that (i) described donkeys deployed as livestock guardians (use), (ii) evaluated LGDk performance, behaviour, or management (*e.g.*, livestock predation outcomes, costs, welfare), or (iii) synthesised such evidence (reviews/guidelines). We excluded sources that mentioned donkeys without a guardian role or that lacked evaluative or synthetic content. The primary focus of the review was literature that was academic, peer-reviewed and published in an academic journal in English. However, given the limited number of peer-reviewed journal articles available on this topic, the search was extended to include grey literature, that was inclusive of government and/or Non-Governmental Organisation (NGO) sources. Sources from agricultural government department databases were incorporated, when deemed relevant to the review. Additionally, NGO reports authored or co-authored by academics in the field were also included, due to valuable insights that bridged the gap between academic research and practical applications. While these reports may not undergo traditional peer review, their inclusion was justified by the expertise and credibility of the authors and the direct relevance of their findings to the research question.

Literature was initially filtered using two search engines: Google Scholar and Web of Science, with no date or geographic limits. The primary Boolean string used in both was: (“donkey” OR “burro” OR “mule” OR “hinny” OR “hinnies”) AND (“livestock guardian” OR “guardian animal” OR “guard” OR “protect”) AND (“livestock” OR “flock” OR “herd”). To capture practice/extension terminology, we also ran targeted variants adding (“conflict mitigation” OR “non-lethal method” OR “carnivore control”). Because LGDk-specific literature is sparse and often older or archived, we used backward snowballing: for each included source we screened its full reference list; we scanned titles using secondary terms (“donkey”, “guard”, “protect”, “herd”, “livestock”), and, when available, checked abstracts or full texts if titles were ambiguous. Several cited items could not be accessed (*n* = 10).

### Literature analysis

Our review included peer-reviewed and grey literature (accessible reports and theses’). For each included source we coded two dimensions: literature type (journal article; thesis/dissertation; government/NGO report or guideline; management document/dataset) and study design (experimental/field trial; observational case study/producer account; social-science survey [interviews/questionnaires]; evidence synthesis/review). We then conducted a comparative narrative synthesis, extracting region, livestock type, focal carnivore taxon, and author-reported LGDk outcomes, and contrasting patterns across regions, taxa, and designs (no meta-analysis due to heterogeneity). We also summarised carnivore species associated with livestock predation where LGDks were used, noting conservation status, ecology, and mitigation methods. For the species-level synthesis we restricted analysis to sources that explicitly linked LGDk use to named carnivore taxa (*n* = 50); broader predation papers without LGDk content were catalogued but not analysed further (*n* = 8).

## Results

### Existing global literature

**Figure 1:**
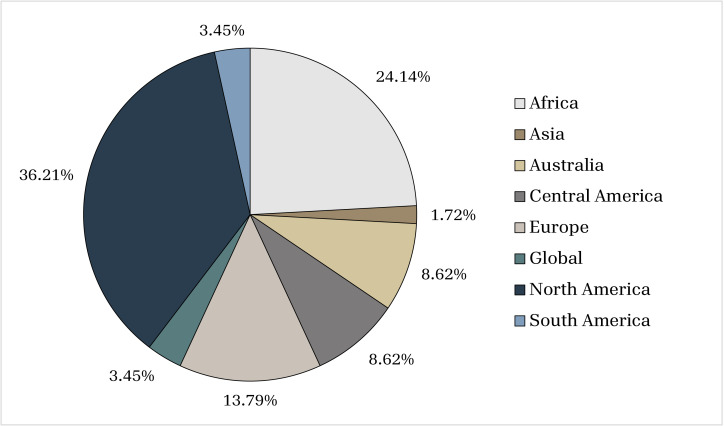
Pie graph depicting proportions and relative percentages of regional literature on LGDk use and effectiveness.

**Figure 2:**
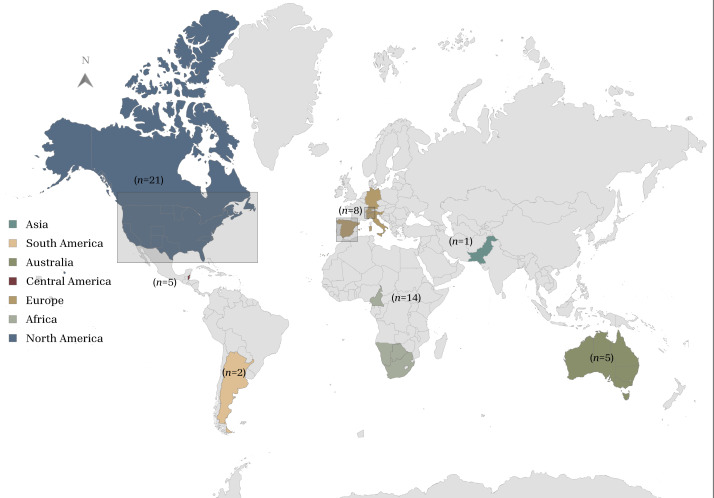
Global distribution of LGDk use by continent in the reviewed literature, excluding global reviews (*n*= 56). Continents are displayed using discrete colours, with labels indicating the number of LGDk-related studies per continent. Shaded regions denote locations where empirical testing of LGDk efficacy has been conducted.

We found a total of 58 peer-reviewed and non-peer reviewed documents that met the criteria to be included in the review. These documents reported on LGDk use in regions of Africa, Asia, Australasia, the Americas (Central, North, and South) and Europe (see Figs. 1 and 2). Out of this sample, two had a global scope and were both reviews (Rigg, 2022; Smith et al., 2000). We also catalogued published criteria/guidelines for LGDk deployment and summarised them in Table 1.

**Table 1:** A summarised table of published criteria for the selection of donkeys to use as livestock guardians (Walton & Feild, 1989).

1. Guard donkeys should be selected from medium to large size stock. Do not use extremely small or miniature donkeys.
2. Do not acquire a donkey which cannot be culled or sold if it fails to perform properly.
3. Use jennies (females) and geldings. Do not use jacks (intact males) as guard animals.
4. Test a new donkey’s guarding response by challenging the donkey with a dog in a corral or small pasture.
5. Use only one donkey or jenny with foal, per pasture.
6. Isolate guard donkeys from horses, mules, and other donkeys.
7. To increase probability of bonding, donkeys should be raised from birth or placed at weaning with sheep and goats.
8. Raise guard donkeys away from dogs. Avoid or limit the use of herding dogs around donkeys.
9. Monitor the use of guard donkeys at lambing or kidding, as some donkeys may be aggressive to newborns or overly possessive. Remove donkeys temporarily if necessary.
10. Use donkeys in small (i.e., 240 ha) open pastures with not more than 200 head of sheep or goats for best results. Large pastures, rough terrain, dense brush, too large a herd, and sheep or goats that are scattered all lessen the effectiveness of guard donkeys.
11. Do not allow donkeys access to feed containing Rumensin, urea or other products intended only for ruminants.

**Figure 3:**
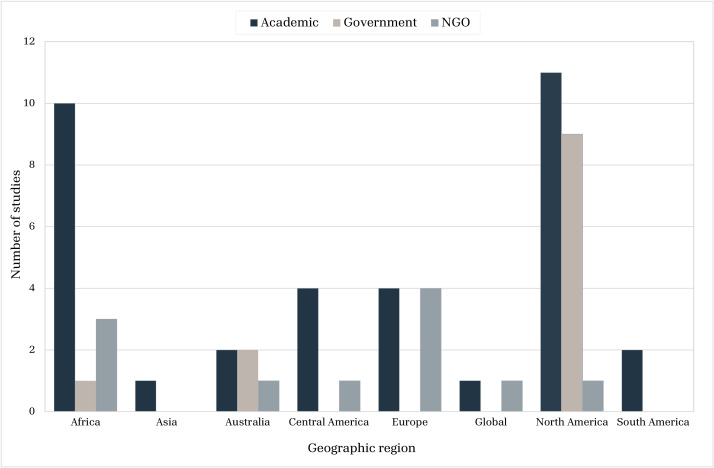
Graph representing publication venue affiliations and geographical distribution of the literature.

As shown in Table 2, the majority of studies were reports (*i.e.,* academic, NGO or government reports, guidelines, manuals or statistical data, etc.), which encompassed over half of the sample (*n* = 32); social science survey-based studies (*n* = 21) were also well represented and included questionnaires (*n* = 7), interviews (*n* = 5), a mixed method of interview-questionnaire (*n* = 6), as well as administrative data collection (*n* = 1) and one study that surveyed agreements between government bodies and producers pertaining to non-lethal method use. Additional studies consisted of three literature reviews and three experimental studies (Table 2). Experimental studies made up 5.1% of the total literature dataset. Affiliations of publication venues were primarily academic (*n* = 35), followed by government departments (*n* = 12) and NGOs (*n* = 11) (see Fig. 3).

**Table 2:** Literature type totals for the collective literature and associated global regions.

Region	Experimental	Social Survey	Report	Review	Total
Africa	–	9	5	–	**14**
Asia	–	–	1	–	**1**
Australia	–	1	4	–	**5**
Central America	–	1	4	–	**5**
Europe	2	2	4	–	**8**
Global	–	–	–	2	**2**
North America	1	6	13	1	**21**
South America	–	2	–	–	**2**
**Total**	**3**	**21**	**31**	**3**	**58**

### Contexts of LGDk use and efficacy

There was notable variation across studies and regions relating to reported efficacy, regional contexts (*i.e.,* biogeography, trends of donkey use), implementation, livestock animals and carnivore species. Livestock species cited in studies on LGDk use were predominantly small stock, with single-species systems (sheep-only or goat-only; *n* = 23) and mixed-species herds (≥2 livestock species; *n* = 18), and to a lesser extent large livestock (*e.g.*, horses, cattle; 13) and poultry (*e.g.*, chickens, turkeys; 2). LGDks were most commonly reported to guard sheep in Australia, Europe, and North America, and cattle in Africa and Central America (see Table 3). Both poultry studies were from North America. Comparisons of individual studies (*n* = 12) selected for their differences in recommended LGDk use, implementation, testing conditions and findings are presented in Table S1. Carnivore species associated with LGDk use in the prevention of carnivore depredation encompassed a total of 27 carnivore species and six carnivore families: *Canidae*, *Felidae*, *Hyaenidae*, *Procyonidae*, *Mustelidae* and *Ursidae*. In-depth examinations into carnivore contexts such as depredation types, conservation status, method use, and conflict mitigation are analysed and presented separately under Appendix A.

**Table 3:** Totals of livestock animal types and their distribution across regions based on the literature sample, excluding global reviews (*n*= 56).

Region	Large stock	Mixed stock	Poultry	Small stock	Total
Africa	6	7	–	1	**14**
Asia	–	1	–	–	**1**
Australia	–	1	–	4	**5**
Central America	4	1	–	–	**5**
Europe	2	–	–	6	**8**
North America	1	7	2	11	**21**
South America	–	1	–	1	**2**
**Total**	**13**	**18**	**2**	**23**	**56**

**Table 4:** Recommendations for LGDk use for various contexts based on collective literature findings and consultations with experts in the field.

Criteria	Context	Recommended use
Terrain	The terrain of an area, such as complexity and steepness, influences LGDk size and effectiveness. For instance, smaller donkeys are better suited to mountainous terrain due to their mobility. Landscape complexity (*e.g.*, open vs. mountainous, vegetation density) affects visibility and carnivore detection.	In areas with steep or challenging terrain, use smaller, more agile donkeys to reduce injury risk and enhance LGDk effectiveness. Consider using multiple LGDks in areas where terrain can obscure visibility or detection of approaching carnivores. Increased herd supervision may also be necessary to minimise risks to both LGDks and livestock.
Training	Studies indicate that while LGDk may not require formal training, foundational training during or after the herd bonding period is essential for optimal performance. This includes improving responsiveness to handlers and encouraging defensive behaviours.	Provide basic training for LGDks to establish strong bonds with the herd, enhance responsiveness to handlers, and encourage effective defensive responses. Processes related to familiarisation and commands can require 2-6 weeks of time investment but are critical for optimising LGDk performance and management.
Sex/Age	Jennies are often preferred due to their ease of handling, management, and stronger carnivore-averse behaviours. Pregnant jennies or those with foals exhibit heightened defensive instincts. Stallions, jacks, and gelded males show variable effectiveness, with some demonstrating increased aggression, particularly during breeding seasons.	Jennies, alone or with foal, are generally more effective due to their strong maternal instincts, increased vigilance and carnivore aversion. However, donkeys should be selected based on individual temperament and specific carnivore context (*e.g.*, species, prevalence, habituation *etc*.,) as a more accurate predictor of LGDk success than sex.
Care and maintenance	Donkeys are perceived as animals that require minimal to no maintenance compared to other guardian animals such as dogs, creating misconceptions about their use and requirements. However, proper care including veterinary attention, adequate nutrition, and basic needs is necessary for both their welfare as well as their efficacy as an LGDk.	Ensure that LGDks receive regular grooming, veterinary care and vaccinations as well as access to adequate resources, such as food, water, shade and shelter. Prioritising proper care improves their longevity and effectiveness in depredation prevention.
Behaviour	Donkeys descended from animals that evolved to travel long distances in harsh environments with limited resources. As a result, donkeys, may exhibit escapist behaviours in search of food and/or mates.	Ensure access to food and water is equal between the LGDk and the herd is equal and synchronised. Providing shared resources (*e.g.*, food, water, housing) helps reduce food-seeking and escapism behaviours. Secure fencing is also essential to prevent LGDks from escaping.
Individual selection	Variability in individual temperaments, particularly in their response to carnivores, significantly influences LGDk success. Not all donkeys exhibit natural aversion towards carnivores, and some may require training to exhibit defensive behaviours.	Test individual donkeys for carnivore-averse temperaments by introducing them to a dog in secure enclosures that are adjacent from one another. Donkeys that exhibit vocal or physical defensive behaviours are more likely to succeed as LGDks compared to those showing fear or indifference.
Carnivore species	The carnivore species targeted by LGDks affects their success, influenced by factors such as carnivore habituation, herd size, and predation behaviours.	Understand the local carnivore species, their predation patterns, and livestock depredation behaviours. Tailor the use of LGDk (*e.g.*, temperament, LGDks used per herd, area size, *etc*.) to the specific context of carnivore behaviour and presence (*e.g.*, habituation, pack size, activity patterns, *etc*.). For greater efficacy, combine LGDks with other non-lethal methods, especially in areas where carnivores are habituated to livestock predation.

## Global use of donkeys as livestock guardians

### North America

Most published criteria for effective LGDk use were derived from producer surveys/reports from 1989–2001, notably Walton & Feild (1989) (see Tables 1 and 4). North American LGDk literature totalled 21 sources (36.2%), spanning Canada, the United States of America (US), and Mexico; designs included academic reports (*n* = 5), social surveys (*n* = 4), one experimental study, and one review, supplemented by government agricultural reports and databases (*n* = 9) as well as one NGO publication. Deployment contexts most often involved small stock (*n* = 11), then mixed herds (*n* = 7), poultry (*n* = 2), and large stock (*n* = 1). Livestock depredation was attributed primarily to canid species, including coyotes, dogs, grey wolves (*Canis lupus*), red foxes (*Vulpes vulpes*) and grey foxes (*Urocyon cinereoargenteus*). Additional incidents involved felids and other carnivores such as pumas (*Puma concolor*), bobcats (*Lynx rufus*), black bears (*Ursus americanus*), brown bears (*Ursus arctos*), raccoons (*Procyon lotor*), and small mustelids (*Mustelidae* spp.) (Andelt, 2004; Beranger, Walters & Botha, 2007; Bourne, 1994; Elkhoraibi et al., 2014; Gese, Hart & Terletzy, 2021; Lance, Primm & Inman, 2023; Larson & Salmon, 1988; May, 1996; Smith et al., 2000; Tischaefer, 2020; United States Department of Agriculture (USDA), 2020; O’Brien, Lansdowne & Cook, 2014; Bergman, Huffman & Paulson, 1998; Walton & Feild, 1989).

#### Studies of LGDk use and efficacy in North America

The study conducted in Texas by Walton & Feild (1989) stands as the most prominent and comprehensive investigation into the utilisation of LGDks to date (Table 1). At the time, Texas held the highest rank in cattle, goat, and sheep production, with livestock predation costs averaging around USD $9 million annually, half of which were attributed to coyotes followed by dogs, foxes, and bobcats (Walton & Feild, 1989).

Two Texas surveys summarised by Walton & Feild (1989) report donkey effectiveness at different sampling levels. In an initial survey of 17 producers covering 58 donkeys, 59% (34/58) of donkeys were rated good or fair. In a subsequent statewide survey sent to 500 producers, 60 producers with donkeys provided carnivore-specific ratings against coyotes (60% fair to excellent) and against dogs (42% fair to excellent). One respondent reported their donkey killing more goats than carnivores, while another described their donkey fending off three coyotes attacking sheep. Green (1989) highlighted the need to pre-test each donkey’s temperament toward dogs before placement, *i.e.,* evaluate whether an individual shows an assertive, dog-averse response rather than indifference or tolerance, because suitability varies widely by temperament. More recently, government reports have noted an increased use of non-lethal methods since the early 2000s, including LGDk use (United States Department of Agriculture (USDA), 2020; United States Department of Agriculture (USDA), 2022). In regions such as Colorado, LGDk use has been actively promoted through government-led initiatives that recognise their effectiveness in reducing livestock depredation, including the donation of wild burros to ranchers to protect cattle herds from wolf predation (Colorado Parks and Wildlife (CPW), 2022).

Trials with donkeys guarding sheep were conducted in Texas and Wyoming. In Texas, effectiveness was low where coyote density and livestock predation were high; there are reports of donkeys fleeing from bears and pumas, and larger carnivores (pumas, grey wolves, bears) may prey on donkeys (Green, 1989; Wilbanks, 1995). A recent empirical pilot in the U.S. tested four feral BLM donkeys with sheep against coyotes and dogs; integration into separate pastures/flocks was achieved by around 5 weeks, outcomes varied with individual and pasture complexity, but vigilance was consistently high (Scasta et al., 2024).

### Central America

The regions covered by the five papers from Central America include Belize and Costa Rica. The literature from Central America primarily consisted of reports (*n* = 4) and one social science survey. Publications were primarily affiliated with academic institutions (*n* = 4) and one from an NGO. Four out of the five reports focused on large stock (cattle, horses), while one addressed mixed stock. Livestock predation involved carnivores such as jaguars (*Panthera onca*), pumas, dogs, and smaller felids like jaguarundis (*Herpailurus yagouaroundi*), ocelots (*Leopardus pardalis*), and margays (*Leopardus wiedii*) (Briggs et al., 2011; Foster, 2008; Harvey, Briggs-Gonzalez & Mazzotti, 2017; Hoogesteijn & Hoogesteijn, 2014; Quigley et al., 2015). Habitats studied included savannahs, tropical lowlands, and cleared farmland.

#### Studies of LGDk use and efficacy in Central America

Evidence is sparse in English sources. In northern Belize, a conservation-payment study found 84.6% of participants owned equids (donkeys/mules/horses), with some adopting donkeys as guardians (Harvey, Briggs-Gonzalez & Mazzotti, 2017). Ranch-level pilots reported by Quigley et al. (2015) show six cattle ranches monitored for 15 months after deploying guard animals (including donkeys) plus improved fencing recorded zero jaguar predation (ranch records, camera traps); interventions were bundled, so donkey-specific effects are not isolated (not tested in Costa Rica due to cost). Foster (2008) notes risks to dog LGAs (jaguars implicated in dog mortality near settlements; dogs detected in jaguar diet only around 3% inside contiguous forest), which may motivate considering donkeys in some contexts. Practical guidance recommends donkeys as alarm-based deterrents and paired with bells and fencing (Briggs et al., 2011; Hoogesteijn & Hoogesteijn, 2014). Overall, LGDk evidence from Central America remains preliminary.

### South America

Two papers (3.4%) were based in South America. They were both conducted in Argentina, specifically, in Buenos Aires Province and Espinal. Both papers were authored by academics and focused on social science surveys related to small livestock (1) or mixed stock (1). Sheep were the main source of income for producers in this region (Luengos Vidal et al., 2016). The habitat types in South America included deciduous woodlands, dense shrublands and grassland prairies, with landscapes mostly characterised as flat and having a semiarid climate. Both papers also noted land modification for agricultural purposes in these areas. Papers from South America related to predation by pumas.

#### Studies of LGDk use and efficacy in South America

English-language documentation is limited and centred on central Argentina. In a Wild Felid Monitor note, only 1 of 7 ranchers using non-lethal measures reported employing a guard donkey, whilst the others relied on night corralling or reinforced enclosures (Luengos Vidal et al., 2016). A broader interview study from the same region emphasised nocturnal corralling (often with dogs) and improved husbandry but did not quantify donkey uptake (Guerisoli et al., 2017). Across these sources, LGDks are grouped with dogs and no study isolates LGDk-specific efficacy. In a small producer sample (*n* = 12), 58.3% reported applying mitigation techniques, with one producer (8.3%) using a donkey (Luengos Vidal et al., 2016). Reported constraints include limited technical support and cost concerns; broader language or institutional factors may also limit reporting and uptake (Guerisoli et al., 2017).

### Europe

In Europe, there were a total of eight papers included in the review, comprising 13.8% of the sample. The literature covered various European countries with studies predominantly focusing on Spain, Slovenia, Switzerland, and Germany. The studies employed different methodologies, including social science surveys (*n* = 2), experimental studies (*n* = 2), and reports (*n* = 4). Four of the studies were produced by academia, while the others were from NGOs related to conservation of both carnivores and rare livestock breeds (*n* = 4). Despite promising reports from both experimental studies, LGDk use was limited, with guard dogs being the preferred choice, likely attributed to their historical use for herd protection across Europe (Van Liere et al., 2013; Angst, Hagen & Breitenmoser, 2002; De Gabriel et al., 2022; Landry, 2000; Reinhardt et al., 2012; Van Bommel & Johnson, 2023).

Most studies involved sheep and goats (*n* = 6), with one study each on horses (*n* = 1) and cattle (*n* = 1). Carnivores associated with LGDk use include brown bear, grey wolf, Eurasian lynx *(Lynx lynx*), grey foxes, red foxes, and dogs.

#### Studies of LGDk use and efficacy in Europe

Evidence of LGDk efficacy has been reported against grey wolves, dogs, foxes and lynx (Van Liere et al., 2013; Angst, Hagen & Breitenmoser, 2002; De Gabriel et al., 2022; Kugler, Grunenfelder & Broxham, 2008; Kugler, 2013; Landry, 2000; Reinhardt et al., 2012). In a pilot experiment in Spain, one Zamorano–Leonese donkey was placed per farm (*n* = 4; herds 20–50 cattle) following Walton & Feild (1989) criteria farmer-reported losses over 12 months before *vs.* after deployment showed a significant decline in predation events (De Gabriel et al., 2022). An observational note from Switzerland describes vigilance/braying and deterrence of canids by donkeys, including two cases of dogs killed while harassing sheep, but does not quantify incident reduction (Landry, 2000). Effectiveness varied with terrain steepness, carnivore habituation/species and individual behaviour, with most behavioural issues involving male donkeys (geldings, jacks, stallions). Collectively, these studies indicate substantial potential, particularly against lynx and dogs, while underscoring the need for further controlled evaluations to optimise deployment and manage behaviour (Angst, Hagen & Breitenmoser, 2002; De Gabriel et al., 2022; Kugler, 2013; Landry, 2000; Reinhardt et al., 2012; Walton & Feild, 1989).

### Australia

Literature focused on LGDks in Australia accounted for 8.6% of the reviewed papers, totalling 5 papers. Reports sourced from government departments (*n* = 2), academic institutions (*n* = 2) and one from an NGO constituted LGDk literature in this region. Australia has observed a significant shift toward non-lethal livestock protection methods, particularly guardian dogs, although there has been limited attention to other LGAs, such as donkeys (P Gibbs, pers. comms., 2024 and D Jenkins, pers. comms., 2024; Bough, 2016; Van Bommel & Johnson, 2023).

Free-ranging donkey populations persist in parts of Australia following 19th–20th century introductions (Bough, 2011; Clancy et al., 2021) but LGDk deployments in our sample were primarily in sheep systems, with some use for calves in dingo/wild-dog areas (Jenkins, 2003). Habitat types conducive to LGDk use were predominantly arid to moderately vegetated, with relatively flat topography which enabled a clear line of sight. Carnivore species implicated in livestock depredation include foxes, dingoes, and dogs (Boronyak & Quartermain, 2022; McLeod, 2016).

#### Studies of LGDk use and efficacy in Australia

LGDks have been reported effective in some contexts against dingoes/wild dogs and red foxes in Australia, and against jackals and caracals in southern Africa (Ballard & Wilson, 2019; Bough, 2016; Du Plessis et al., 2018; Jenkins, 2003). A prominent paper cited across global contexts is a government report by Jenkins (2003) outlining various LGAs suitable for Australian farmers and presenting case study reports from farmers. The report highlighted promising evidence of LGDks efficacy in reducing livestock depredation, particularly by dingoes and/or dogs. Additional studies outlined cases of successful use of captured wild donkeys as LGDks (Bough, 2016). This study identified key welfare considerations and emphasised the critical importance of accessible training and guidance to support livestock producers in the responsible and effective deployment of LGDks.

### Africa

Our sample included 14 sources (24.1%) from Namibia, Botswana and South Africa (with references to Ethiopia/Kenya/Zimbabwe), mostly social-science surveys (*n* = 9) and reports (*n* = 5) (Drouilly, Nattrass & O’Riain, 2023); Du Plessis et al., 2018; (Horgan, 2015; Humphries, Hill & Downs, 2015; Marker-Kraus et al., 1996; Schumann, 2009; Viollaz, Thompson & Petrossian, 2021). Deployments were chiefly in mixed herds (*n* = 7) and cattle systems (*n* = 6), with one small-stock study. Producer reports from South Africa during LGDk use noted lamb/kid (sheep/goat) weaning rates of 91–100% and small-stock predation by jackals and caracals of 0–10% (Botha, 2018). In Namibia, one producer reported reducing calf losses from 32 in 1986 to near zero after adopting donkeys (Marker-Kraus et al., 1996). Several sources recommend LGDks principally for jackals and caracals, often alongside other measures (*e.g.*, kraaling, guard dogs) (Drouilly, Nattrass & O’Riain, 2023; Du Plessis et al., 2018; Horgan, 2015; Humphries, Hill & Downs, 2015; Luyt, 2020). Overall, evidence is observational or grey literature, implemented alongside other interventions, making it difficult to isolate the effect of donkeys alone. Quantitative evaluations remain scarce, and although larger carnivores are mentioned, LGDk-specific testing is limited (Horgan, 2015; Karidozo, La Grange & Osborn, 2016; Schumann, 2009; Smuts, 2008; Viollaz, Thompson & Petrossian, 2021).

#### Studies of LGDk use and efficacy in Africa

Reports of LGDk efficacy in this region were primarily observational or anecdotal, derived from social science surveys conducted with livestock producers. Reports of LGDk efficacy largely document reductions in livestock predation by jackals, caracals, dogs, leopards (*Panthera pardus*) and cheetahs (*Acinonyx jubatus*) (Botha, 2018; Karidozo, La Grange & Osborn, 2016; Marker-Kraus et al., 1996; Schumann, 2009; Schumann, Watson & Schumann, 2008; Thorn et al., 2012). Producer reports from South Africa indicated improved outcomes while LGDks were in use: lamb/kid (sheep/goat) weaning rates of 91–100% and small-stock predation losses to jackals and caracals of 0–10% (survey/observational data; (Botha, 2018). Likewise, a Namibian producer reported reducing calf losses from 32 animals in 1986 to near zero after adopting LGDks (Marker-Kraus et al., 1996).

Additional reports recommended the use of LGDks for preventing depredation by lions (*Panthera leo*), brown hyenas (*Parahyaena brunnea*), spotted hyenas (*Crocuta crocuta*) and African painted dogs (*Lycaon pictus*) (Horgan, 2015; Karidozo, La Grange & Osborn, 2016; Marker-Kraus et al., 1996; Schumann, 2004; Smuts, 2008). Examples include reported use of two LGDks per cattle herd having apparent success in Kenya to prevent depredation by lions (Schumann, 2004; Smuts, 2008). In South Africa, farmers reported that LGDks ‘worked well’ against leopards, with one farmer reporting that an enraged LGDk can even kill a leopard by kicking it with its hooves, echoing other reports of leopards being trampled to death in Namibia (Marker-Kraus et al., 1996; Viollaz, Thompson & Petrossian, 2021). Conversely, lions, leopards, and spotted hyenas have been reported to predate on donkeys in some regions (Du Plessis et al., 2018; Luyt, 2020). Predation is particularly common when donkeys are left tied or neglected (Camillo et al., 2018; Humphries, Hill & Downs, 2015; Karidozo, La Grange & Osborn, 2016). In some areas, donkeys are also used for other purposes, such as baiting large carnivores for trophy hunting or research (see Fig. S1) which can complicate interpretation of predation events.

### Asia

Across Asia, in countries including India, Nepal, Bhutan, Mongolia, Pakistan, Afghanistan, Thailand and China, donkeys are used for transport, military roles, milk/meat production, and other tasks (Kubasiewicz et al., 2022; Khan et al., 2013; Maheshwari & Sathyakumar, 2020; Norris et al., 2021; Seyiti & Kelimu, 2021; Singh, 2022; Todd et al., 2022; Wang et al., 2022). However, only one paper in our sample examined donkey use in Asia (1.7%; Khan et al., 2013).

#### Studies of LGDk use and efficacy in Asia

Across Asia, documentation of LGDks is sparse. We found only brief mentions from Pakistan of donkeys guarding cattle, goats, and sheep, with no target carnivores specified and no quantitative outcomes reported (Khan et al., 2013). Beyond these notes, we found no studies that directly evaluate LGDk deployment, mechanisms, or efficacy in Asian systems; overall, LGDk research from Asia remains limited in our sample (Norris et al., 2021).

## Carnivore species associated with depredation and LGDk use

### Carnivore species and conservation status

**Figure 4:**
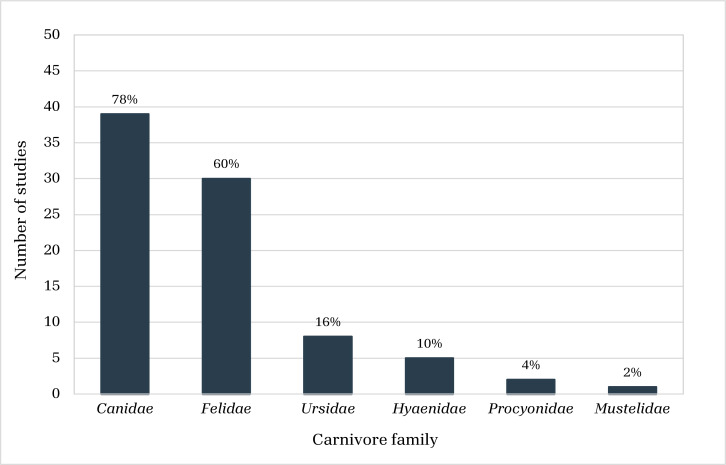
Proportions and relative percentages of all six carnivore families represented across carnivore-specific literature related to LGDk use to prevent carnivore depredation events (*n*= 50).

Of the 58 studies, 50 explicitly named carnivore taxa associated with LGDk use. Across these sources, LGDks were used against 27 species in six families; coverage was dominated by *Canidae* (39 papers; 78%), most often dogs, coyotes, red foxes, grey wolves, black-backed jackals, followed by *Felidae* (30 papers; 60%; commonly pumas, caracals, bobcats, cheetahs, leopards). Mentions of *Hyaenidae, Ursidae*, *Procyonidae*, and *Mustelidae* were infrequent (Fig. 4). We note that studies often reported LGDks alongside other measures, but here we summarise only the carnivores linked to LGDk deployments. Most wild species named are listed as ‘Least Concern’ under International Union for Conservation of Nature (IUCN). Red List of Threatened Species, with a minority listed as ‘Near Threatened’, ‘Vulnerable’, or ‘Endangered’ (International Union for Conservation of Nature (IUCN), 2025). Detailed conservation statuses are provided in Appendix A (Table A1).

## Comparative analysis with popular LGAs as a non-lethal method

### Dogs

**Figure 5:**
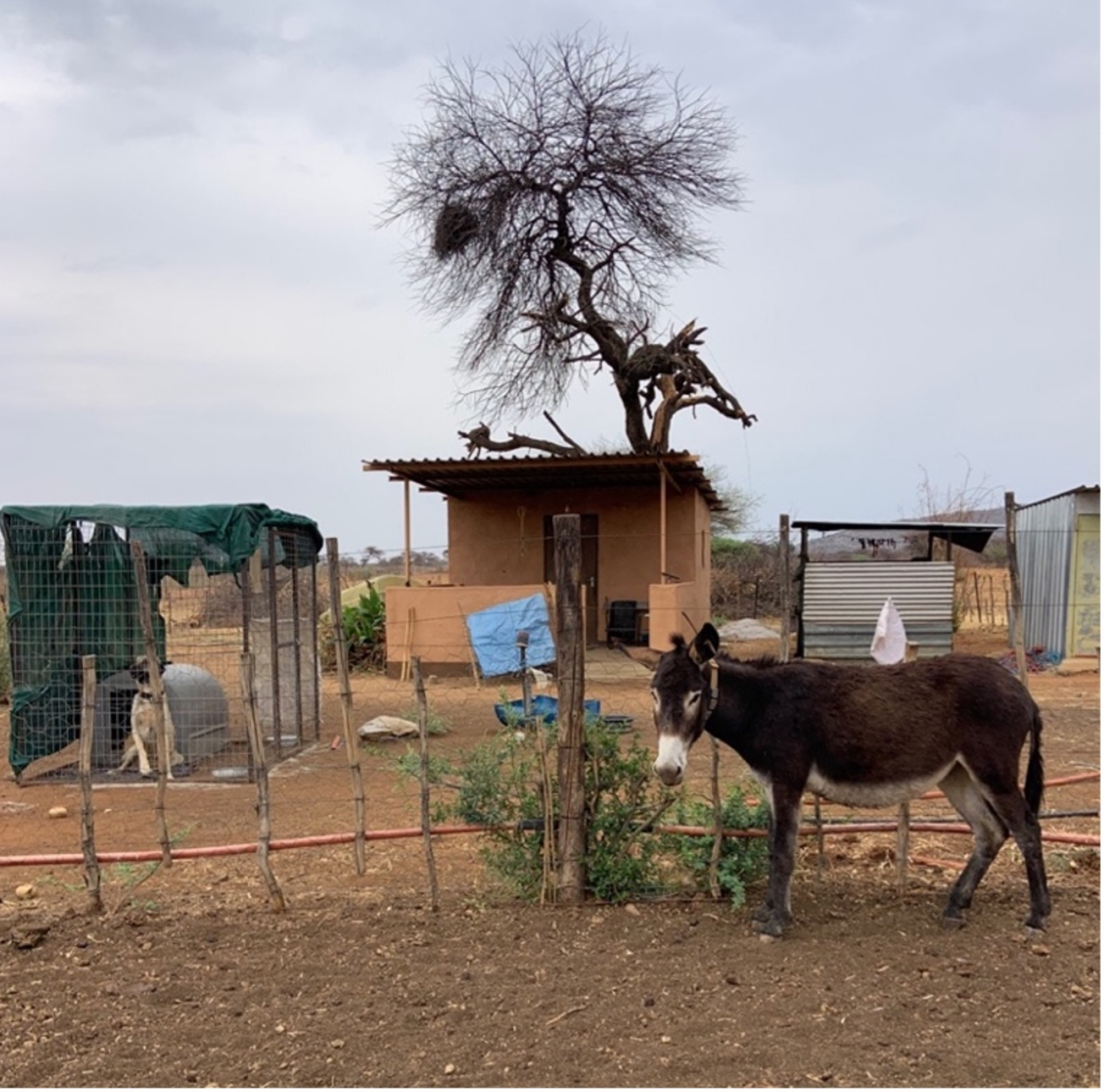
An LGDk housed with an Anatolian Shepherd for combined use in guarding roles in northwest Namibia. Shared use of space between LGAs and their protected herds is recommended as crucial for familiarisation and the bonding process Additionally, familiarisation between LGAs should occur prior to herd integration and encompass periods of both active work and off-duty rest. This process is particularly important for mitigating canid-averse behaviours toward non-threatening canids that may reside on the same farm. Photo taken by first author.

Livestock guardian dogs (LGDs) have a long history and, when well selected, trained and managed, can substantially reduce livestock predation by small and large carnivores, including coyotes, wolves, pumas, bears, leopards, cheetahs, dingoes and foxes (Bangs et al., 2006; Coppinger & Coppinger, 2000; Drouilly et al., 2020; Landry, Borelli & Drouilly, 2020; Linnell & Lescureux, 2015; Marker-Kraus et al., 1996; Rigg et al., 2011; Van Der Weyde et al., 2020). Performance varies with landscape and husbandry; dogs can cover large grazing areas, whereas management in small, subdivided pastures may be more challenging (Coppinger & Coppinger, 2000). Breed differences are reported and can inform farmer choice (Coppinger & Coppinger, 2014). Pairing dogs with donkeys is sometimes recommended to combine early alarm/deterrence with close-in confrontation (Fig. 5) (Landry, 2000; Marker-Kraus et al., 1996; Walton, 1990).

Costs and risks are non-trivial: LGDs require housing, veterinary care and feed; some individuals harass wildlife or show aggression toward people if poorly managed (De Gabriel et al., 2022; Drouilly et al., 2020; Gehring, VerCauteren & Cellar, 2011; Smith et al., 2000). Wild carnivores may treat LGDs as intruders or competitors and attack or kill them (*e.g.*, wolf–dog conflicts in Europe and North America) (Bangs et al., 2006; Gehring, VerCauteren & Cellar, 2011; Gula, 2008; Landry, Borelli & Drouilly, 2020). Comparable risks exist for donkeys where large carnivores may attack or kill LGDks (Green, 1989; Wilbanks, 1995). In summary, LGDs and LGDks offer complementary strengths: dogs are suited to wide-ranging herds with intensive handler input; donkeys may fit smaller, open pastures with primarily canid threats and lower ongoing costs, but both require appropriate selection, training and oversight for welfare and effectiveness.

### Llamas

**Figure 6:**
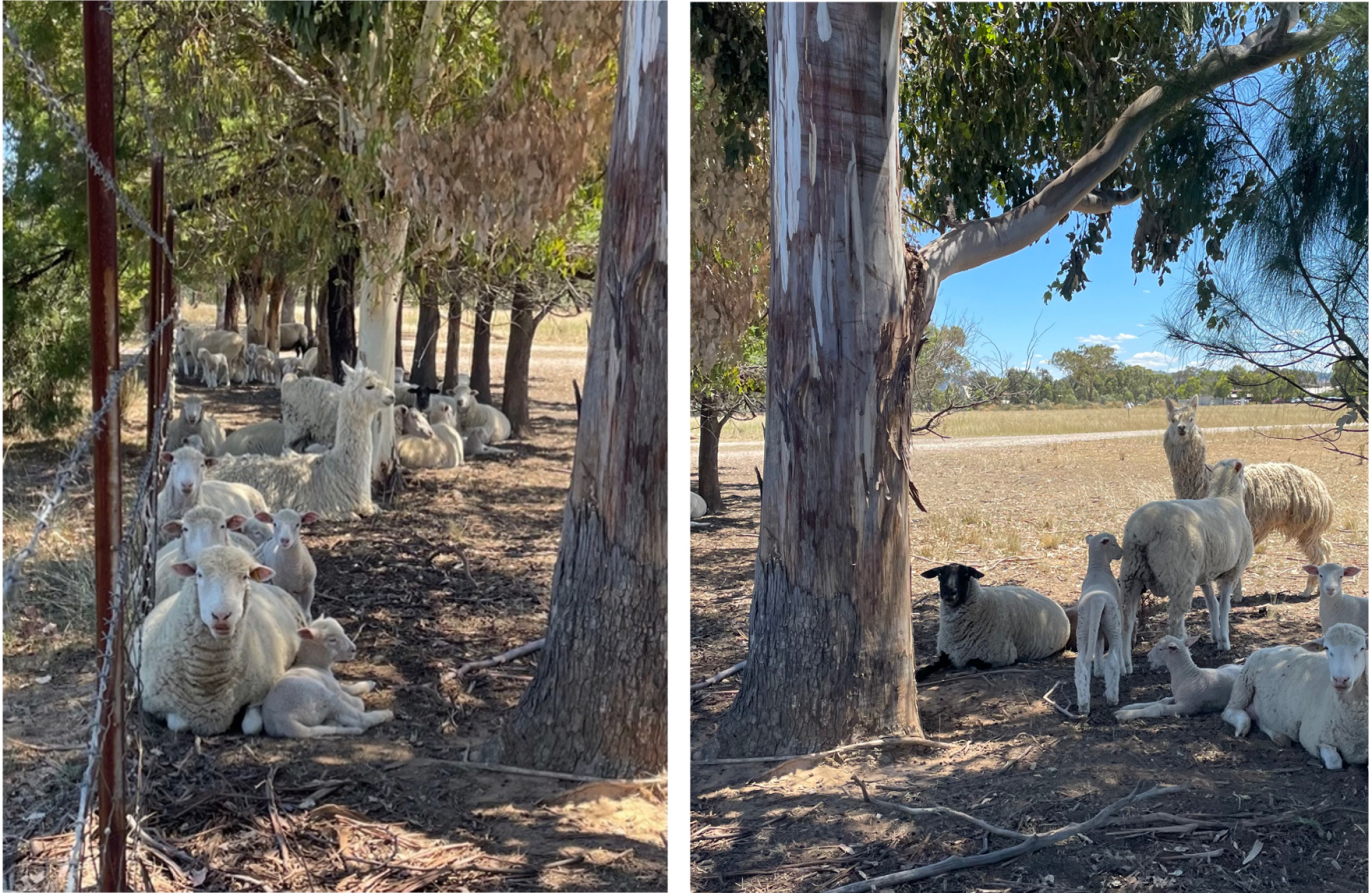
Two llamas guard a flock of sheep comprised mostly of ewes and lambs in the central western region of New South Wales, Australia. Photo taken by first author.

Llamas are used as livestock guardians, especially with sheep (Fig. 6), and, like donkeys, show canid-averse behaviours (alarm calling, approach, chase) with reports of effectiveness against coyotes and other small canids (Andelt, 2004; Franklin, Powell & Youngs, 1994; Franklin & Powell, 2006). Compared with donkeys, published evidence for direct defence against larger carnivores (*e.g.*, pumas, bears) is limited or cautions reduced effectiveness; llamas are typically framed as alarm or deterrence guardians rather than physical confrontation, whereas donkeys more often engage at close range (Franklin, Powell & Youngs, 1994; Gese, Hart & Terletzy, 2021) (see also Green, 1989; Smith et al., 2000). Effectiveness for packs of canids may be lower for llamas (Andelt, 2004; Franklin, Powell & Youngs, 1994).

Practical considerations also differ. Llamas bond readily with sheep and often require minimal training to stay with the flock but can be scarce and entail higher purchase/maintenance costs than donkeys in some regions; occasional behavioural issues (*e.g.*, mounting attempts) require oversight (Andelt, 2004; Franklin, Powell & Youngs, 1994; Gese, Hart & Terletzy, 2021; Luyt, 2020). In multi-method systems, dogs and donkeys/llamas are sometimes combined to pair early alarm with close-in deterrence (Green, 1989; Landry, 2000; Marker-Kraus et al., 1996; Walton, 1990).

## Discussion

### Summary of the evidence base

Across the 58 sources we reviewed, most evidence on LGDks is observational or grey literature, with very few experimental evaluations. Reported deployments are concentrated in North America and southern Africa, with scattered records elsewhere. LGDks are used primarily with small stock (sheep, goats), with additional reports from mixed herds and, less commonly, cattle systems. Threat profiles are dominated by canids (coyotes, free-ranging dogs, foxes and, in some cases, wolves), with fewer studies addressing felids or other carnivore families (*e.g.*, pumas, caracals, lynx). Where outcomes were quantified, producers frequently reported reductions in livestock predation following LGDk adoption, however, interventions were often bundled (*e.g.*, corralling, fencing, dogs used concurrently), so donkey-specific effects were seldom isolated (*e.g.*, Walton & Feild, 1989; Smith et al., 2000; Landry, 2000; Jenkins, 2003; De Gabriel et al., 2022; Scasta et al., 2024; Botha, 2018; Marker-Kraus et al., 1996).

### LGDk deployment contexts and performance (with caveats)

Several practice patterns emerge from the literature. LGDks are most often reported in small, open pastures, where vigilance, alarm braying, and direct approach or pursuit can deter canids (Bourne, 1994; Gese, Hart & Terletzy, 2021; Green, 1989; D Jenkins, pers. comm., 2024; Landry, 2000; Ontario Ministry of Agriculture, Food and Rural Affairs (OMAFRA), 2020; Scasta et al., 2024), although proper bonding with a herd may allow effectiveness over larger distances (Green, 1989; P. Gibbs, pers. comm., 2024). Secondly, individual selection and management are critical: jennies or geldings are generally preferred over intact males, and a short, structured bonding period is consistently recommended (Green, 1989; Jenkins, 2003; Ontario Ministry of Agriculture, Food and Rural Affairs (OMAFRA), 2020; Walton & Feild, 1989). Thirdly, context dependence is clear: steep/complex terrain, very high coyote densities, or the presence of larger carnivores are associated with reduced reliability, including instances of donkeys fleeing or being injured (Green, 1989; Wilbanks, 1995). Together, these patterns suggest that LGDks are best framed as non-lethal deterrents suited to canid-driven systems in relatively open country, ideally stacked with basic husbandry (*e.g.*, fencing, supervised turnout).

### Signals from stronger study designs

Where experimental or quasi-experimental designs were used, results are promising but preliminary. In Spain, a pilot study placing one Zamorano-Leonese donkey per cattle farm (*n* = 4) reported a significant decline in predation events in the 12 months after deployment relative to the 12 months prior (De Gabriel et al., 2022). In a recent U.S. pilot with four feral BLM donkeys guarding sheep, integration took around 5 weeks and vigilance was consistently high, although outcomes varied with donkey individuality and pasture complexity (Scasta et al., 2024). In Switzerland, an observational note documented deterrence behaviours and two incidents of dogs killed while harassing sheep, but no quantitative reduction was reported (Landry, 2000). In southern Africa, producer reports during LGDk use noted lamb/kid weaning 91–100% and small-stock losses 0–10% to jackals and caracals (Botha, 2018), and a Namibian case described reducing calf losses from 32 in 1986 to near zero after adopting donkeys (Marker-Kraus et al., 1996). These accounts indicate potential but also underline the need for donkey-specific effect sizes under controlled designs.

### Comparison with other guardian species (dogs and camelids)

LGDs can be highly effective across large grazing areas when well managed but entail higher ongoing husbandry costs and risks (*e.g.*, wildlife conflicts, aggressive behaviour if poorly socialised) (Bangs et al., 2006; Coppinger & Coppinger, 2000; Coppinger & Coppinger, 2014; Drouilly et al., 2020; Gehring, VerCauteren & Cellar, 2011; Rigg et al., 2011). Llamas are often framed as alarm and deterrent guardians for small canids and integrate easily with sheep but may be less reliable with packs or larger carnivores (Andelt, 2004; Franklin, Powell & Youngs, 1994; Franklin & Powell, 2006; Smith et al., 2000). Donkeys sit between these: generally lower ongoing costs than dogs, stronger close-range deterrence than llamas, and best suited to canid threats in open country, however, they share vulnerability to larger carnivores and require appropriate selection and bonding (Green, 1989; Jenkins, 2003; Landry, 2000).

### Risks and failure modes

Common failure modes include poor match between donkey temperament and threat profile, use of intact males, inadequate bonding, and deployment in terrain where donkeys cannot maintain proximity or visibility to stock (Angst, Hagen & Breitenmoser, 2002; Green, 1989; Jenkins, 2003; Landry, 2000; Walton & Feild, 1989). Large carnivores can also attack or kill donkeys, particularly where carnivore densities are high or where animals are placed singly (Green, 1989; Marker-Kraus et al., 1996; Ontario Ministry of Agriculture, Food and Rural Affairs (OMAFRA), 2020). Misuse, such as deploying donkeys in areas with multiple or habituated carnivores, increases the risk of injury and reduced effectiveness in preventing depredation (Foster, 2008; Gehring, VerCauteren & Cellar, 2011; Gula, 2008).

### Evidence gaps and research priorities

The literature would benefit from: (i) donkey-specific before *vs.* after or treatment-control designs that report absolute loss rates and confidence intervals; (ii) standardised metrics (clear event definitions; denominators such as stock-days or minimum monitoring periods); (iii) analysis of context moderators (terrain/visibility, carnivore density, fencing, herd size/stocking rate, sex/age/temperament, bonding protocol); (iv) coverage in under-represented regions and threat profiles; and (v) practical economics and welfare (up-front and ongoing costs relative to dogs/llamas; stock and donkey wellbeing indicators) (*e.g.*, Walton & Feild, 1989; Smith et al., 2000; Landry, 2000; Jenkins, 2003; Eklund et al., 2017; Treves, Krofel & McManus, 2016; Van Bommel & Johnson, 2023; De Gabriel et al., 2022; Scasta et al., 2024; Botha, 2018).

### Interim guidance for practice

Based on current evidence, three practice elements appear most consistently associated with positive outcomes: (i) selection of jennies or geldings with dog-averse temperament; (ii) a short, structured bonding period prior to turnout; and (iii) combining LGDks with basic husbandry measures such as fencing, foundational training and additional non-lethal method use (De Gabriel et al., 2022; Green, 1989; Jenkins, 2003; Ontario Ministry of Agriculture, Food and Rural Affairs (OMAFRA), 2020; Walton & Feild, 1989).

## Ethical Management and Maintenance of LGDks

**Figure 7:**
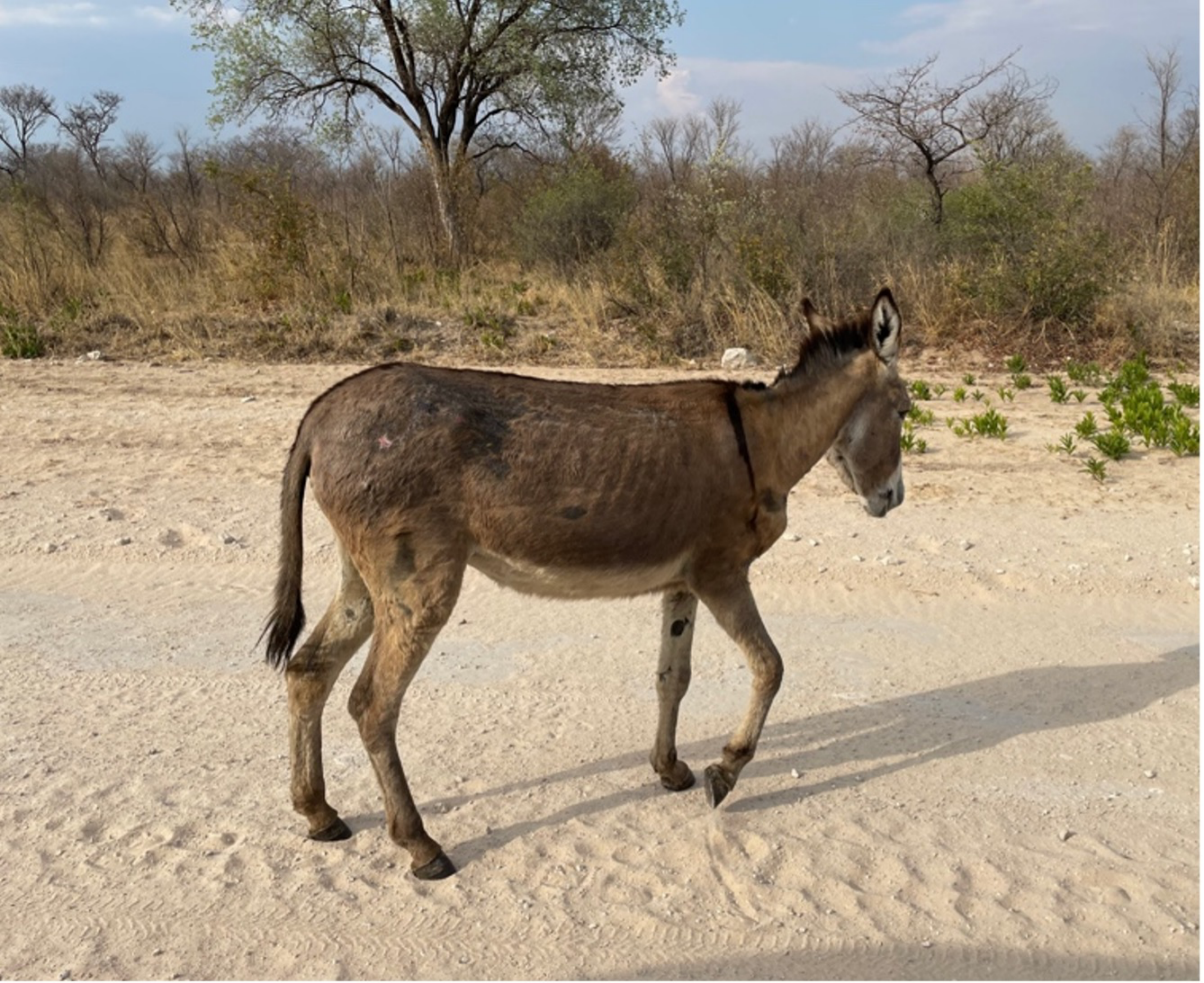
An individual free-roaming donkey exhibiting poor body condition and welfare observed in a conservancy area, northeastern Namibia. This picture was taken along a main road in a conservancy area in northeastern Namibia. In conservancy areas, it is common for livestock, including donkeys, belonging to nearby villages to roam freely, as fences are not permitted within these areas. The donkey depicted here was alone and exhibited signs of malnourishment, along with injuries likely resulting from saddle or cart use. Photo taken by first author.

Beyond performance-related risks, ethical deployment of LGDks requires sustained attention to animal welfare and responsible management. Although LGDks typically require less maintenance than other guardian animals, regular care remains essential to ensure both welfare and efficacy. Ethical concerns arise when ineffective LGDks are abandoned, neglected, or culled, underscoring the need for informed decision-making, responsible deployment, and ongoing monitoring (Braithwait, 1996; Walton & Feild, 1989). Safeguarding animal welfare therefore requires the establishment of clear selection criteria, minimum care standards, and explicit protocols for removal or rehoming where appropriate (see Table 4) (De Gabriel et al., 2022; Smith et al., 2000). Essential care includes foundational training and structured bonding with livestock, access to feed, water, and shelter, regular exercise, hoof trimming, vaccinations, and routine health checks (Botha, 2018; Bough, 2011; Jenkins, 2003; McLean & Gonzalez, 2018; P. Gibbs, pers. comm., 2024). Donkeys’ stoic nature can obscure welfare issues, and improper management can lead to malnutrition, obesity, disease, or injury (Burden & Thiemann, 2015; Camillo et al., 2018; McLean & Gonzalez, 2018). Ethical concerns also arise from historical misuse of donkeys as beasts of burden and from inadequate care in LGDk deployments, including injuries during transport or capture, insufficient veterinary attention, and poor living conditions (Fig. 7) (Botha, 2018; Bough, 2011; Bough, 2016; Camillo et al., 2018; Regan et al., 2014). These factors highlight the importance of informed, consistent management to safeguard both welfare and guardian effectiveness.

## Sociocultural and economic considerations

Donkeys used as livestock guardians offer a low-cost, sustainable means of protecting livestock, particularly valuable in rural or low-income regions vulnerable to predation losses. However, affordability and accessibility vary globally, with prices rising significantly, sometimes four- to tenfold, due to the international demand for donkey skins for ejiao production (Seyiti & Kelimu, 2021; Quigley et al., 2015). Adoption barriers also include entrenched social norms, competition with lethal control methods, limited policy support, and low awareness stemming from scarce research and testing (Boronyak, Jacobs & Smith, 2023; Drouilly, Nattrass & O’Riain, 2023; Eklund et al., 2017; Horgan, 2015; Jenkins, 2003; Schumann, Watson & Schumann, 2008).

Cultural beliefs and gender dynamics strongly influence donkey ownership and use (Ayad et al., 2019; Geiger et al., 2020; Harvey, Briggs-Gonzalez & Mazzotti, 2017). In Ethiopia and other countries such as Ghana, Mexico, and India, donkeys are vital for women’s economic empowerment (Geiger et al., 2020; Maggs, Ainslie & Bennett, 2021; Ravichandran et al., 2023; Rodrigues et al., 2021). Conversely, in parts of North Africa, traditional norms restrict donkey use by gender, forbidding jennies for labour (Ayad et al., 2019). Effective HCC mitigation strategies, including LGDk programs, must therefore integrate local beliefs and cultural contexts. Research on LGDks remains limited in both English and non-English publications (Foster, 2008).

## Global Trends in Current and Future Donkey Use

Globally, donkeys serve diverse roles, from livestock guardians, therapy animals, crop protectors and transportation (Camillo et al., 2018; Norris et al., 2021; Musyoki, 2014; Schepers, 2016). More recent use relates to the surge in demand for ejiao a gelatin made from donkey skins used in traditional Chinese medicine, has driven severe welfare and conservation crises (Cathcart, 2024; Johnston, 2024; Seyiti & Kelimu, 2021). China’s donkey population fell from 11 million in 1990 to 6 million in 2013 due to domestic consumption of around 4 million skins annually (The Donkey Sanctuary, 2019). This depletion has shifted sourcing to regions such as Africa, Latin America, and the Middle East, often affecting marginalised rural communities. For instance, Peru exported 208 tons of skins illegally between 2015–2016, while Mexico now exports about 10 million skins annually (Johnston, 2024; The Donkey Sanctuary, 2019).

While initially perceived as economically beneficial, the ejiao trade has destabilised rural economies and exacerbated welfare concerns (Cathcart, 2024; Johnston, 2024; Bennett & Pfuderer, 2020). In response, over fifteen African and South American nations including Uganda, Tanzania, Botswana, Niger, and Brazil, have imposed export bans or restrictions since 2015 (Seyiti & Kelimu, 2021; The Donkey Sanctuary, 2019). The U.S. is also pursuing legislative prohibition in various regions (The Donkey Sanctuary, 2019). The intersection of escalating trade demand, population collapse, and minimal research on LGDks highlights an urgent need for policy and scientific action to safeguard both donkeys and the communities that depend on them.

## Conclusion

Our review identified 58 documents on the use and efficacy of donkeys as livestock guardians to prevent or minimise carnivore depredation. Only 5.1% of studies were experimental, whilst the remainder comprised social-science surveys and reviews affiliated with academia (31%) and NGOs (5.1%), academic reports (22%), government reports/databases (22%), other NGO sources (9%), and review-based literature (5.1%). Collectively, the limited empirical validation highlights the need for peer-reviewed, donkey-specific tests of LGDk efficacy under controlled or clearly defined before-and-after designs.

Reported outcomes and limitations varied by region, study design, livestock type, and carnivore guild. Most deployments involved small stock and canid threats, with performance appearing context-dependent: positive results were most often associated with appropriate individual selection (typically jennies or geldings), basic foundational training, a short, structured bonding period, and integration with basic husbandry (*e.g.*, fencing, supervised turnout). Reduced reliability was noted in steep/complex terrain, guarding too large herds or paddocks, and areas with high carnivore density. These patterns indicate that LGDks are best considered a targeted, non-lethal tool whose effectiveness depends on matching deployment to context and management.

Major barriers to wider adoption and evaluation include limited donkey-specific effect sizes, heterogeneous outcome metrics, and inconsistent reporting of denominators (*e.g.*, stock-at-risk) and monitoring duration. Future research should prioritise: (i) donkey-specific experimental or before-and-after studies reporting absolute loss rates with uncertainty; (ii) standardised metrics (event definitions, stock-days, minimum monitoring periods); (iii) tests of context moderators (terrain/visibility, fencing, herd structure, sex/age/temperament, bonding protocol); and (iv) practical costs and welfare considerations relevant to producers.

Pending such evidence, LGDks can play a useful role within broader conflict-mitigation portfolios, particularly in canid-driven systems and smaller, open pastures, provided that selection, bonding and husbandry protocols are in place to support both effectiveness and animal welfare.

## Supplemental Information

10.7717/peerj.21265/supp-1 Supplemental Information 1 Supplementary Files *Note.* Selected sample of studies from the literature sample relating to donkeys either as recommended (rec.) or implemented LGDk use. Studies were chosen to demonstrate the discrepancies and differences between regions, recommendations, use and reports of efficacy. Abbreviations in the first column represent the study design and were observational (Obs), report (Rep) or experimental (Exp). To indicate the efficacy of LGDks involved in a study a symbol was used to depict which studies found LGDks effective (✓) or ineffective (x).

10.7717/peerj.21265/supp-2 Supplemental Information 2 Literature Dataset - Livestock Guardian Donkeys (LGDks)

## Appendix

**Table A1:** Carnivore taxa linked to reported use of donkeys as livestock guardian animals (LGDks), showing IUCN global conservation status, instances where LGDks were reported as protective or recommended for deterring those taxa (with sources), and instances where negative outcomes were reported, including donkey predation or ineffectiveness (with sources). Conservation status codes follow the IUCN Red List (LC = Least Concern, NT = Near Threatened, VU = Vulnerable, EN = Endangered). Symbols indicate evidence availability rather than definitive outcomes;  denotes taxa with multiple (≥3) published reports of LGDk use or perceived effectiveness; –indicates no record in the reviewed literature;  denotes cases where evidence was minimal or not tested in the available sources. Additional rows summarise, for each taxon, reported depredation types (small stock, large stock, poultry, pets, infrastructure damage) and mitigation methods recorded alongside LGDk use (lethal, passive non-lethal, active non-lethal, other non-lethal deterrents, compensation). These entries reflect only information reported in LGDk-linked sources and do not constitute a comprehensive review or definitive assessment of LGDk effectiveness for each species.

Species	IUCN status	LGDk protective use reported	Literature	LGDk failure or predation reported	Literature	Depredation type	Mitigation method
**African painted dog** (*Lycaon pictus*)	EN		Horgan, 2015; Smuts, 2008	**–**			
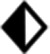
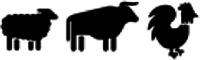
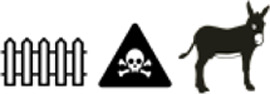
**Black bear** (*Ursus americanus*)	LC		Bourne, 1994		Andelt, 2004; Macon et al., 2018		
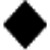
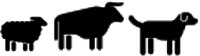
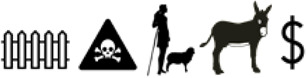
**Black-backed jackal** (*Lupulella mesomelas*)	LC		Botha, 2018; Marker-Kraus et al., 1996; Smuts, 2008	**–**			
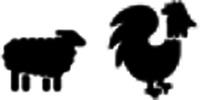
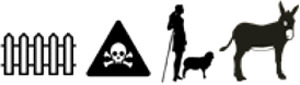
**Bobcat** (*Lynx rufus*)	LC		Beranger, Walters & Bender, 2007; Green, 1989; Macon et al., 2018; Walton & Feild, 1989	**–**	Wilbanks, 1995		
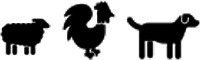
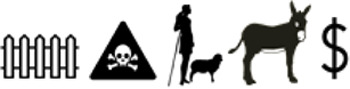
**Brown bear** (*Ursus arctos*)	LC		Bourne, 1994		Macon et al., 2018; Wilbanks, 1995		
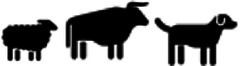
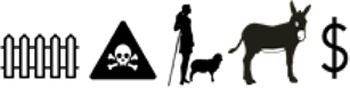
**Brown hyena** (*Parahyaena brunnea*)	NT		Karidozo, La Grange & Osborn, 2016	–			
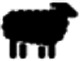
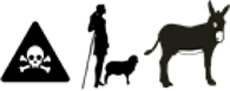
**Cape fox** (*Vulpes chama*)	LC		Drouilly, Nattrass & O’Riain, 2023	**–**			
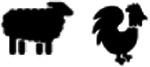
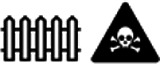
**Caracal** (*Caracal caracal*)	LC		Botha, 2018; Marker-Kraus et al., 1996; Schumann, 2009; Smuts, 2008		Drouilly, Nattrass & O’Riain, 2023		
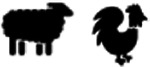
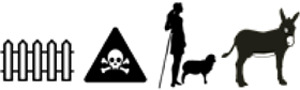
**Cheetah** (*Acinonyx jubatus*)	VU		Karidozo, La Grange & Osborn, 2016; Luyt, 2020; Marker-Kraus et al., 1996; Schumann, 2009; Smuts, 2008	**–**			
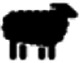
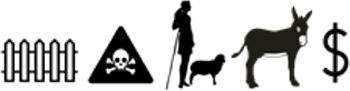
**Coyote** (*Canis latrans*)	LC		Bourne, 1994; Green, 1989; Larson & Salmon, 1988; Scasta et al., 2024; Walton & Feild, 1989	**–**	Green, 1989		
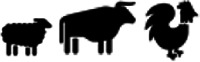
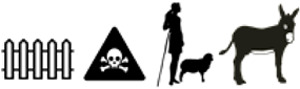
**Dingo** (*Canis lupis dingo*)	N/A		Boronyak, Jacobs & Smith, 2023; Bough, 2016; D Jenkins, pers. comm., 2024; P. Gibbs, pers. comm. 2024		Du Plessis et al., 2018		
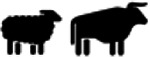
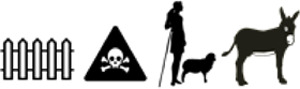
**Domestic cat** (*Felis familiaris*)	N/A		Beranger, Walters & Bender, 2007	**–**			
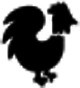
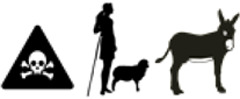
**Domestic dog** (*Canis familiaris*)	N/A		Bourne, 1994; Braithwait, 1996; De Gabriel et al., 2022; Green, 1989; Landry, 2000; Marker-Kraus et al., 1996; Scasta et al., 2024; Walton & Feild, 1989	**–**			
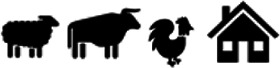
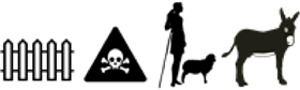
**Eurasian lynx** (*Lynx lynx*)	LC		Angst, Hagen & Breitenmoser, 2002; Reinhardt et al., 2012	**–**			
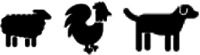
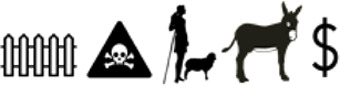
**Grey fox** (*Urocyon cinereoargenteus*)	LC		Green, 1989	**–**			
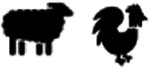
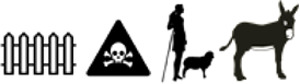
**Grey wolf** (*Canis lupis*)	LC		De Gabriel et al., 2022; Colorado Parks and Wildlife (CPW), 2022; Gese, Hart & Terletzy, 2021; Kugler, 2013; Lance, Primm & Inman, 2023		Gese, Hart & Terletzy, 2021; Green, 1989;		
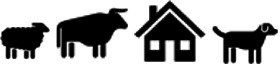
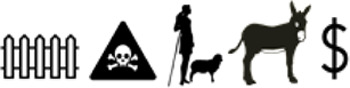
**Jaguar** (*Panthera onca*)	NT		Briggs et al., 2011; Foster, 2008; Hoogesteijn & Hoogesteijn, 2014; Quigley et al., 2015	**–**			
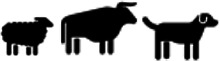
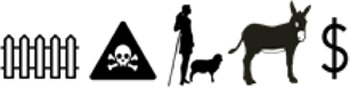
**Jaguarundi** (*Herpailurus yagouaroundi*)	LC		Briggs et al., 2011; Harvey, Briggs-Gonzalez & Mazzotti, 2017	**–**			
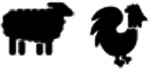
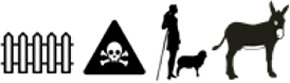
**Leopard** (*Panthera pardus*)	VU		Marker-Kraus et al., 1996; Schumann, Watson & Schumann, 2008; Schumann, 2009; Smuts, 2008; Viollaz, Thompson & Petrossian, 2021		Du Plessis et al., 2018; Luyt, 2020		
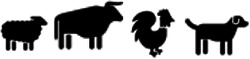
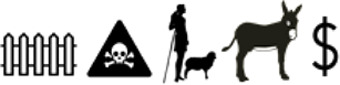
**Lion** (*Panthera leo*)	VU		Schumann, 2004; Smuts, 2008		Du Plessis et al., 2018; Karidozo, La Grange & Osborn, 2016		
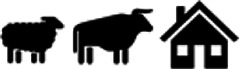
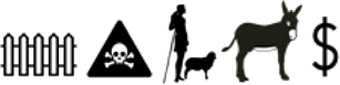
**Margay** (*Leopardus wiedii*)	NT		Briggs et al., 2011; Harvey, Briggs-Gonzalez & Mazzotti, 2017	**–**			
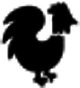
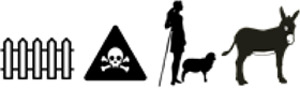
**Ocelot** (*Leopardus pardalis*)	LC		Briggs et al., 2011; Harvey, Briggs-Gonzalez & Mazzotti, 2017	**–**			
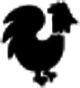
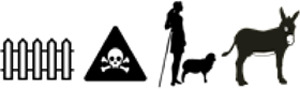
**Puma** (*Puma concolor*)****	LC		Briggs et al., 2011; Larson & Salmon, 1988; Quigley et al., 2015		Green, 1989; Macon et al., 2018; Wilbanks, 1995		
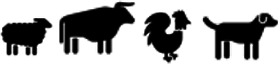
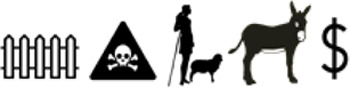
**Raccoon** (*Procyon lotor*)	LC		Beranger, Walters & Bender, 2007; Larson & Salmon, 1988	**–**			
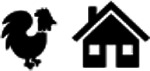
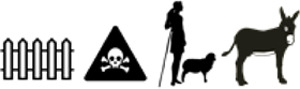
**Red fox** (*Vulpes vulpes*) ****	LC		Bough, 2016; De Gabriel et al., 2022; Green, 1989; Jenkins, 2003; Walton & Feild, 1989	**–**			
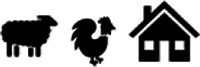
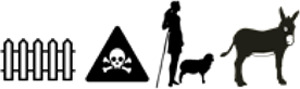
**Small Mustelids** (*Mustelidae* spp.)	LC		Beranger, Walters & Bender, 2007	**–**			
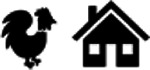
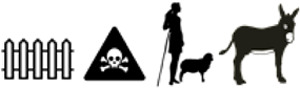
**Spotted hyena** (*Crocuta crocuta*)	LC		Horgan, 2015; Schumann, Watson & Schumann, 2008		Du Plessis et al., 2018		
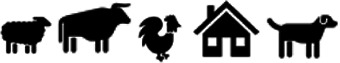
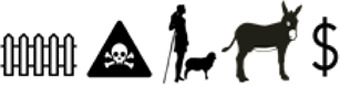

**Notes.**

**Damage associated with carnivore species**.

–Predation of poultry (chickens, turkeys).

–Predation of large livestock (cattle, equids).

–Predation of small livestock (goats, sheep).

–Predation of domestic pets.

–Damage to infrastructure (*e.g.*, housing, roofing, fences, enclosures *etc*.,)

**Mitigation methods related to carnivore depredation**

–Lethal methods (*e.g.*, poison, baiting, shooting, gin traps, snares, fire, hunting dogs, *etc*.).

–Passive non-lethal methods (*e.g.*, physical barriers, exclusion fencing, enclosures, bomas or kraals, *etc*.).

–Active non-lethal methods (*e.g.*, shepherd supervision, GPS radio-collars, geometric fencing, electric fencing, *etc*.).

–Non-lethal deterrents (*e.g.*, guardian animals, the use of visual, olfactory and/or auditory deterrents such as fladry, fox lights, firecrackers, *etc*.).

–Compensation payments for losses incurred due to carnivore depredation.

## Additional Information and Declarations

### Data Availability

The following information was supplied regarding data availability: This is a literature review.

## References

[ref-1] Andelt WF (2004). Use of livestock guarding animals to reduce predation on livestock. Sheep & Goat Research Journal.

[ref-2] Angst C, Hagen S, Breitenmoser U (2002). Attacks by lynxes on small livestock and enclosed animals in Switzerland: part II—measures to protect farm animals (KORA Report No. 10.

[ref-3] Asa CS, Marshall F, Fischer M (2012). Affiliative and aggressive behaviour in a group of female Somali wild ass (*Equus africanus somalicus*). Zoo Biology.

[ref-4] Ayad A, Aissanou S, Amis K, Latreche A, Iguer-Ouada M (2019). Morphological characteristics of donkeys (*Equus asinus*) in Kabylie area, Algeria. Slovak Journal of Animal Science.

[ref-5] Ballard JWO, Wilson LA (2019). The Australian dingo: untamed or feral?. Frontiers in Zoology.

[ref-6] Bangs E, Jimenez M, Niemeyer C, Fontaine J, Collinge M, Krsichke R, Handegard L, Shivik J, Sime C, Nadeau S, Mack C, Smith DW, Asher V, Stone S (2006). Non-lethal and lethal tools to manage wolf-livestock conflict in the Northwestern United States.

[ref-7] Bennett R, Pfuderer S (2020). The potential for new donkey farming systems to supply the growing demand for hides. Animals.

[ref-8] Beranger J, Walters M, Bender M (2007). Protecting heritage turkeys from carnivores. How to raise heritage turkeys on pasture.

[ref-9] Bergman DL, Huffman LE, Paulson JD (1998). North Dakota’s cost-share program for guard animals. https://digitalcommons.unl.edu/vpc18/31.

[ref-10] Boronyak L, Jacobs B, Smith B (2023). Unlocking lethal dingo management in Australia. Diversity.

[ref-11] Boronyak L, Quartermain E (2022). Carnivore smart farming: modernising Australia’s approach to livestock protection.

[ref-12] Botha J (2018). A survey of farmers’ experience using guard animals to control the impact of carnivores on farm livestock. Master’s thesis.

[ref-13] Bough J (2011). Donkey.

[ref-14] Bough J (2016). Our stubborn prejudice about donkeys is shifting as they protect Australia’s sheep from wild dogs. Australian Zoologist.

[ref-15] Bourne J (1994). Protecting livestock with guard donkeys. Agri-Facts.

[ref-16] Braczkowski AR, O’Bryan CJ, Lessmann C, Rondinini C, Crysell AP, Gilbert S, Stringer M, Gibson L, Biggs D (2023). The unequal burden of human-wildlife conflict. Communications Biology.

[ref-17] Braithwait J (1996). Using guard animals to protect livestock.

[ref-18] Breitenmoser U, Angst C, Landry JM, Breitenmoser-Würsten C, Linnell JDC, Weber J-M, Woodroffe R, Thirgood S, Rabinowitz A (2005). Non-lethal techniques for reducing depredation. People and wildlife, conflict or co-existence?.

[ref-19] Briggs VS, Harvey RG, Mazzotti FJ, Giuliano WM (2011). A guide to living with wild cats: WEC314/UW359, 10/2011. EDIS.

[ref-20] Burden F, Thiemann A (2015). Donkeys are different. Journal of Equine Veterinary Science.

[ref-21] Camillo F, Rota A, Biagini L, Tesi M, Fanelli D, Panzani D (2018). The current situation and trend of donkey industry in Europe. Journal of Equine Veterinary Science.

[ref-22] Cathcart C (2024). Ejiao and the donkey skin trade: an urgent one health concern. The Canadian Veterinary Journal.

[ref-23] Clancy CL, Kubasiewicz LM, Raw Z, Cooke F (2021). Science and knowledge of free-roaming donkeys—a critical review. The Journal of Wildlife Management.

[ref-24] Colorado Parks and Wildlife (CPW) (2022). CPW donates burros to help North Park rancher prevent further wolf depredations.

[ref-25] Coppinger L, Coppinger R (2000). Guarding livestock. Livestock handling and transport.

[ref-26] Coppinger L, Coppinger R (2014). Dogs for herding and guarding livestock. Livestock handling and transport.

[ref-27] De Gabriel J, Talegón J, Casas V, Ribeiro S (2022). Trials of Zamorano-Leonese donkeys to protect cattle. Carnivore Damage Prevention News.

[ref-28] Di Minin E, Slotow R, Hunter LTB, Montesino Pouzols F, Toivonen T, Verburg PH, Leader-Williams N, Petracca L, Moilanen A (2016). Global priorities for national carnivore conservation under land use change. Scientific Reports.

[ref-29] Dohner JV (2007). Livestock guardians: using dogs, donkeys, and llamas to protect your herd.

[ref-30] Drouilly M, Kelly C, Cristescu B, Teichman KJ, Riain MJ (2020). Investigating the hidden costs of livestock guarding dogs: a case study in Namaqualand, South Africa. Journal of Vertebrate Biology.

[ref-31] Drouilly M, Nattrass N, O’Riain MJ (2023). Small-livestock farmers’ perceived effectiveness of predation control methods and the correlates of reported illegal poison use in the South African Karoo. Ambio.

[ref-32] Du Plessis J, Avenant N, Botha A, Mkhize N, Müller L, Mzileni N, O’Riain M, Parker D, Potgieter G, Richardson P, Rode S, Viljoen N, Hawkins HJ, Tafani M, Kerley G, Wilson S, Balfour B (2018). Past and current management of predation on livestock. Livestock predation and its management in South Africa: a scientific assessment.

[ref-33] Eklund A, López-Bao JV, Tourani M, Chapron G, Frank J (2017). Limited evidence on the effectiveness of interventions to reduce livestock predation by large carnivores. Scientific Reports.

[ref-34] Elkhoraibi C, Blatchford RA, Pitesky ME, Mench JA (2014). Backyard chickens in the United States: a survey of flock owners. Poultry Science.

[ref-35] Foster R (2008). The ecology of jaguars (Panthera onca) in a human-influenced landscape. Doctoral dissertation.

[ref-36] Franklin W, Powell K (2006). Guard llamas: a part of integrated sheep protection.

[ref-37] Franklin WL, Powell KJ, Youngs CR (1994). Guard llamas.

[ref-38] Gehring TM, VerCauteren KC, Cellar AC (2011). Good fences make good neighbors: implementation of electric fencing for establishing effective livestock-protection dogs. Human-Wildlife Interactions.

[ref-39] Geiger M, Hockenhull J, Buller H, Tefera Engida G, Getachew M, Burden FA, Whay HR (2020). Understanding the attitudes of communities to the social, economic, and cultural importance of working donkeys in rural, peri-urban, and urban areas of Ethiopia. Frontiers in Veterinary Science.

[ref-40] Gese EM, Hart JP, Terletzy PA (2021). Gray Wolves. Wildlife damage management technical series.

[ref-41] Green JS (1989). Donkeys for predation control.

[ref-42] Guerisoli MLM, Luengos Vidal E, Franchini M, Caruso N, Casanave EB, Lucherini M (2017). Characterization of puma-livestock conflicts in rangelands of central Argentina. Royal Society Open Science.

[ref-43] Gula R (2008). Wolf depredation on domestic animals in the Polish Carpathian Mountains. The Journal of Wildlife Management.

[ref-44] Harvey RG, Briggs-Gonzalez V, Mazzotti FJ (2017). Conservation payments in a social context: determinants of tolerance and behavioural intentions towards wild cats in northern Belize. Oryx.

[ref-45] Hoogesteijn R, Hoogesteijn A (2014). Anti-predation strategies for cattle ranches in Latin America: a guide. Panthera.

[ref-46] Horgan JE (2015). Testing the effectiveness and cost-efficiency of livestock guarding dogs in Botswana. Doctoral dissertation.

[ref-47] Humphries BD, Hill TR, Downs CT (2015). Landowners’ perspectives of black-backed jackals (*Canis mesomelas*) on farmlands in KwaZulu-Natal, South Africa. African Journal of Ecology.

[ref-48] Inskip C, Zimmermann A (2009). Human-felid conflict: a review of patterns and priorities worldwide. Oryx.

[ref-49] International Union for Conservation of Nature (IUCN) (2025). The IUCN Red list of threatened species.

[ref-50] Janeiro-Otero A, Newsome TM, Van Eeden LM, Ripple WJ, Dormann CF (2020). Grey wolf (*Canis lupus*) predation on livestock in relation to prey availability. Biological Conservation.

[ref-51] Jenkins DJ (2003). Guard animals for livestock protection: existing and potential use in Australia.

[ref-52] Johnston LA (2024). China, Africa, and the market for Donkeys: why did the African Union Ban the *Ejiao*-Linked Donkey hide trade?. Journal of Contemporary China.

[ref-53] Karidozo M, La Grange M, Osborn FV (2016). Assessment of the human wildlife conflict mitigation measures being implemented by the Kavango-Zambezi Transfrontier Conservation Area (KAZA TFCA) partner countries. Report to the KAZA TFCA Secretariat (BMZ No.: 2009 66 788 & BMZ No.: 2006 65 646).

[ref-54] Khan MS, Shah MGU, Shah SAH, Gandahiz JA, Khan SA, Alam F, Lochi GM, Hasan SM (2013). Donkey traction, use and welfare needs at Daman region of Dera Ismail Khan, Pakistan. Scientific Research and Essays.

[ref-55] Kubasiewicz L, Watson T, Norris S, Chamberlain N, Nye C, Perumal R, Saroja R, Raw Z, Burden F (2022). One welfare: linking poverty, equid ownership and equid welfare in the brick kilns of India. Animal Welfare.

[ref-56] Kugler W (2013). Added value of Donkey breeds in Europe Project Report 2013.

[ref-57] Kugler W, Grunenfelder HP, Broxham E (2008). Donkey breeds in Europe.

[ref-58] Lance N, Primm S, Inman K (2023). Wolf resource guide: hands-on resource guide to reduce depredations.

[ref-59] Landry JM (2000). Testing livestock guard donkeys in the Swiss Alps. Carnivore Damage Prevention News.

[ref-60] Landry JM, Borelli JL, Drouilly M (2020). Interactions between livestock guarding dogs and wolves in the southern French Alps. Journal of Vertebrate Biology.

[ref-61] Larson S, Salmon TP (1988). Carnivores and sheep management practices in Sonoma County, California.

[ref-62] Lazure L, Weladji RB (2024). Methods to mitigate human–wildlife conflicts involving common mesocarnivores: a meta-analysis. The Journal of Wildlife Management.

[ref-63] Linnell JD, Lescureux N (2015). Livestock guarding Dogs—cultural heritage icons with a new relevance for mitigating conservation conflicts.

[ref-64] Lõoke M, Mongillo P, Bortoletti M, Normando S, Marinelli L (2024). Characterization of social behavior in a group of domestic donkeys (*Equus asinus*). Applied Animal Behaviour Science.

[ref-65] Luengos Vidal EM, Guerisoli MDLM, Caruso N, Franchini M, McDonald Z, Lucherini M (2016). Characterization and mitigation of puma-livestock conflicts in central Argentina. Wild Felid Monitor.

[ref-66] Luyt EDC (2020). Farming with Carnivores—an agroecological approach to human-wildlife conflict on Namibian farmlands. Doctoral dissertation.

[ref-67] Macon D, Baldwin R, Lile D, Stackhouse J, Rivers CK, Saitone T, Schohr T, Snell L, Harper J, Ingram R, Rodrigues K, Macaulay L, Roche L (2018). Livestock protection tools for California ranchers.

[ref-68] Maggs HC, Ainslie A, Bennett RM (2021). Donkey ownership provides a range of income benefits to the livelihoods of rural households in Northern Ghana. Animals.

[ref-69] Maheshwari A, Sathyakumar S (2020). Patterns of livestock depredation and large carnivore conservation implications in the Indian Trans-Himalaya. Journal of Arid Environments.

[ref-70] Marker-Kraus L, Kraus D, Barnett D, Hurlbut S (1996). Cheetah survival on Namibian farmlands.

[ref-71] May JA (1996). Results of a non-lethal survey and report provided to the New Mexico legislature.

[ref-72] McLean AK, Gonzalez FJN (2018). Can scientists influence donkey welfare? Historical perspective and a contemporary view. Journal of Equine Veterinary Science.

[ref-73] McLeod R (2016). Cost of pest animals in NSW and Australia, 2013-14. Report prepared for the NSW Natural Resources.

[ref-74] McManus JS, Dickman AJ, Gaynor D, Smuts BH, Macdonald DW (2015). Dead or alive? Comparing costs and benefits of lethal and non-lethal human–wildlife conflict mitigation on livestock farms. Oryx.

[ref-75] Murray LM, Byrne K, D’Eath RB (2013). Pair-bonding and companion recognition in domestic donkeys, Equus asinus. Applied Animal Behaviour Science.

[ref-76] Musyoki C (2014). Crop defense and coping strategies: wildlife raids in Mahiga’B’village in Nyeri District, Kenya. African Study Monographs.

[ref-77] Naha D, Dash SK, Chettri A, Chaudhary P, Sonker G, Heurich M, Rawat GS, Sathyakumar S (2020). Landscape predictors of human-leopard conflicts within multi-use areas of the Himalayan region. Scientific Reports.

[ref-78] Norris SL, Little HA, Ryding J, Raw Z (2021). Global donkey and mule populations: figures and trends. PLOS ONE.

[ref-79] O’Brien A, Lansdowne B, Cook M (2014). Predation management with a focus on coyotes.

[ref-80] Ontario Ministry of Agriculture, Food and Rural Affairs (OMAFRA) (2020). Guidelines for using donkeys as guard animals with sheep.

[ref-81] Quigley H, Hoogesteijn R, Hoogesteijn A, Foster R, Payan E, Corrales D, Salom-Perez R, Urbina Y (2015). Observations and preliminary testing of jaguar depredation reduction techniques in and between core jaguar populations. Parks.

[ref-82] Ravichandran T, Perumal RK, Vijayalakshmy K, Raw Z, Cooke F, Baltenweck I, Rahman H (2023). Means of livelihood, clean environment to women empowerment: the multi-faceted role of donkeys. Animals.

[ref-83] Regan FH, Hockenhull J, Pritchard JC, Waterman-Pearson AE, Whay HR (2014). Behavioural repertoire of working donkeys and consistency of behaviour over time, as a preliminary step towards identifying pain-related behaviours. PLOS ONE.

[ref-84] Reinhardt I, Rauer G, Kluth G, Kaczensky P, Knauer F, Wotschikowsky U (2012). Livestock protection methods applicable for Germany—a Country newly recolonized by wolves. Hystrix.

[ref-85] Rigg R (2001). Livestock guarding dogs: their current use worldwide.

[ref-86] Rigg R (2022). Are donkeys good livestock guardians?. Carnivore Damage Prevention News.

[ref-87] Rigg R, Finďo S, Wechselberger M, Gorman ML, Sillero-Zubiri C, Macdonald DW (2011). Mitigating carnivore–livestock conflict in Europe: lessons from Slovakia. Oryx.

[ref-88] Ripple WJ, Estes JA, Beschta RL, Wilmers CC, Ritchie EG, Hebblewhite M, Berger J, Elmhagen B, Letnic M, Nelson MP, Schmitz OJ, Smith DW, Wallach AD, Wirsing AJ (2014). Status and ecological effects of the world’s largest carnivores. Science.

[ref-89] Rodrigues JB, Raw Z, Santurtun E, Cooke F, Clancy C (2021). Donkeys in transition: changing use in a changing world. Brazilian Journal of Veterinary Research and Animal Science.

[ref-90] Scasta JD, Stewart WC, Hutchinson E, Koepke KL, De M. Lima PT, Laverell D, Kersh A, Stam B (2024). From wild to watchful: integrating BLM donkeys (burros) for sheep ranch protection. Sheep & Goat Research Journal.

[ref-91] Schepers A (2016). The economic impact of predation in the wildlife ranching industry in Limpopo, South Africa. Doctoral dissertation.

[ref-92] Schumann B (2009). The needs of emerging commercial farmers in Namibia in relation to human-carnivore conflict. Doctoral dissertation.

[ref-93] Schumann EM (2004). Guide to integrated livestock and carnivore management.

[ref-94] Schumann M, Watson LH, Schumann BD (2008). Attitudes of Namibian commercial farmers toward large carnivores: the influence of conservancy membership. South African Journal of Wildlife Research-24-Month Delayed Open Access.

[ref-95] Seyiti S, Kelimu A (2021). Donkey industry in China: current aspects, suggestions and future challenges. Journal of Equine Veterinary Science.

[ref-96] Singh KV (2022). Biodiversity and conservation status of indigenous livestock of India. Biodiversity.

[ref-97] Smith ME, Linnell JDC, Odden J, Swenson JE (2000). Review of methods to reduce livestock depredation: I. Guardian animals. Acta Agriculturae Scandinavica, Section A, Animal Science.

[ref-98] Smuts B (2008). Carnivores on livestock farms: a practical farmers’ manual for non-lethal, holistic, ecologically acceptable and ethical management.

[ref-99] The Donkey Sanctuary (2019). Under the skin: the emerging trade in donkey skins and its implications for donkey welfare and livelihoods.

[ref-100] Thorn M, Green M, Dalerum F, Bateman PW, Scott DM (2012). What drives human–carnivore conflict in the North West Province of South Africa?. Biological Conservation.

[ref-101] Tischaefer R (2020). Coyotes. Wildlife damage management technical series.

[ref-102] Todd ET, Tonasso-Calvière L, Chauvey L, Schiavinato S, Fages A, Seguin-Orlando A, Clavel P, Khan N, Pérez Pardal L, Patterson Rosa L, Librado P, Ringbauer H, Verdugo M, Southon J, Aury J-M, Perdereau A, Vila E, Marzullo M, Prato O, Tecchiati U, Gianni BG, Tagliacozzo A, Tinè V, Alhaique F, Cardoso JL, Valente MJ, Antunes MT, Frantz L, Shapiro B, Bradley DG, Boulbes N, Gardeisen A, Horwitz LK, Öztan A, Arbuckle BS, Onar V, Clavel B, Lepetz S, Vahdati AA, Davoudi H, Mohaseb A, Mashkour M, Bouchez O, Donnadieu C, Wincker P, Brooks SA, Beja-Pereira A, Wu D-D, Orlando L (2022). The genomic history and global expansion of domestic donkeys. Science.

[ref-103] Treves A, Karanth KU (2003). Human-carnivore conflict and perspectives on carnivore management worldwide. Conservation Biology.

[ref-104] Treves A, Krofel M, McManus J (2016). Predator control should not be a shot in the dark. Frontiers in Ecology and the Environment.

[ref-105] Ugarte CS, Moreira-Arce D, Simonetti JA (2019). Ecological attributes of carnivore-livestock conflict. Frontiers in Ecology and Evolution.

[ref-106] Ullah N, Basheer I, Rehman FU, Zhang M, Khan MT, Khan S, Du H (2024). Livestock depredation by large carnivores and human–wildlife conflict in two districts of Balochistan Province, Pakistan. Animals.

[ref-107] United States Department of Agriculture (USDA) (2020). Sheep death loss 2020: sheep and lamb carnivore and noncarnivore death loss in the United States.

[ref-108] United States Department of Agriculture (USDA) (2022). Wolf damage management in Minnesota 2022.

[ref-109] Van Bommel L, Johnson CN (2023). Still a good dog! Long-term use and effectiveness of livestock guardian dogs to protect livestock from carnivores in Australia’s extensive grazing systems. Wildlife Research.

[ref-110] Van Der Weyde LK, Kokole M, Modise C, Mbinda B, Seele P, Klein R (2020). Reducing livestock-carnivore conflict on rural farms using local livestock guarding dogs. Journal of Vertebrate Biology.

[ref-111] Van Eeden LM, Crowther MS, Dickman CR, Macdonald DW, Ripple WJ, Ritchie EG, Newsome TM (2018). Managing conflict between large carnivores and livestock. Conservation Biology.

[ref-112] Van Liere D, Dwyer C, Jordan D, Premik-Banič A, Valenčič A, Kompan D, Siard N (2013). Farm characteristics in Slovene wolf habitat related to attacks on sheep. Applied Animal Behaviour Science.

[ref-113] Viollaz JS, Thompson ST, Petrossian GA (2021). When human-wildlife conflict turns deadly: comparing the situational factors that drive retaliatory leopard killings in South Africa. Animals.

[ref-114] Walton MT (1990). Rancher use of livestock protection collars in Texas.

[ref-115] Walton MT, Feild CA (1989). Use of donkeys to guard sheep and goats in Texas.

[ref-116] Wang Y, Hua X, Shi X, Wang C (2022). Origin, evolution, and research development of Donkeys. Gene.

[ref-117] Wilbanks CA, Rollins D, Richardson C, Blankenship T, Canon K, Henke S (1995). Alternative methods of carnivore control. Coyotes in the Southwest: a compendium of our knowledge.

