# Supplementary material for: A global review of Livestock Guardian Donkeys (LGDks) in the prevention of carnivore depredation
Source: PeerJ. 2026 Jun 24;14:e21265. doi: 10.7717/peerj.21265 (PMC13310045; doi:10.7717/peerj.21265)
Supplement: Supplemental Information 1 — Note. Selected sample of studies from the literature sample relating to donkeys either as recommended (rec.) or implemented LGDk use. Studies were chosen to demonstrate the discrepancies and differences between regions, recommendations, use and reports of efficacy. Abbreviations in the first column represent the study design and were observational (Obs), report (Rep) or experimental (Exp). To indicate the efficacy of LGDks involved in a study a symbol was used to depict which studies found LGDks effective (✓) or ineffective (x). [file peerj-14-21265-s001.docx]

**Supplementary Material**

**
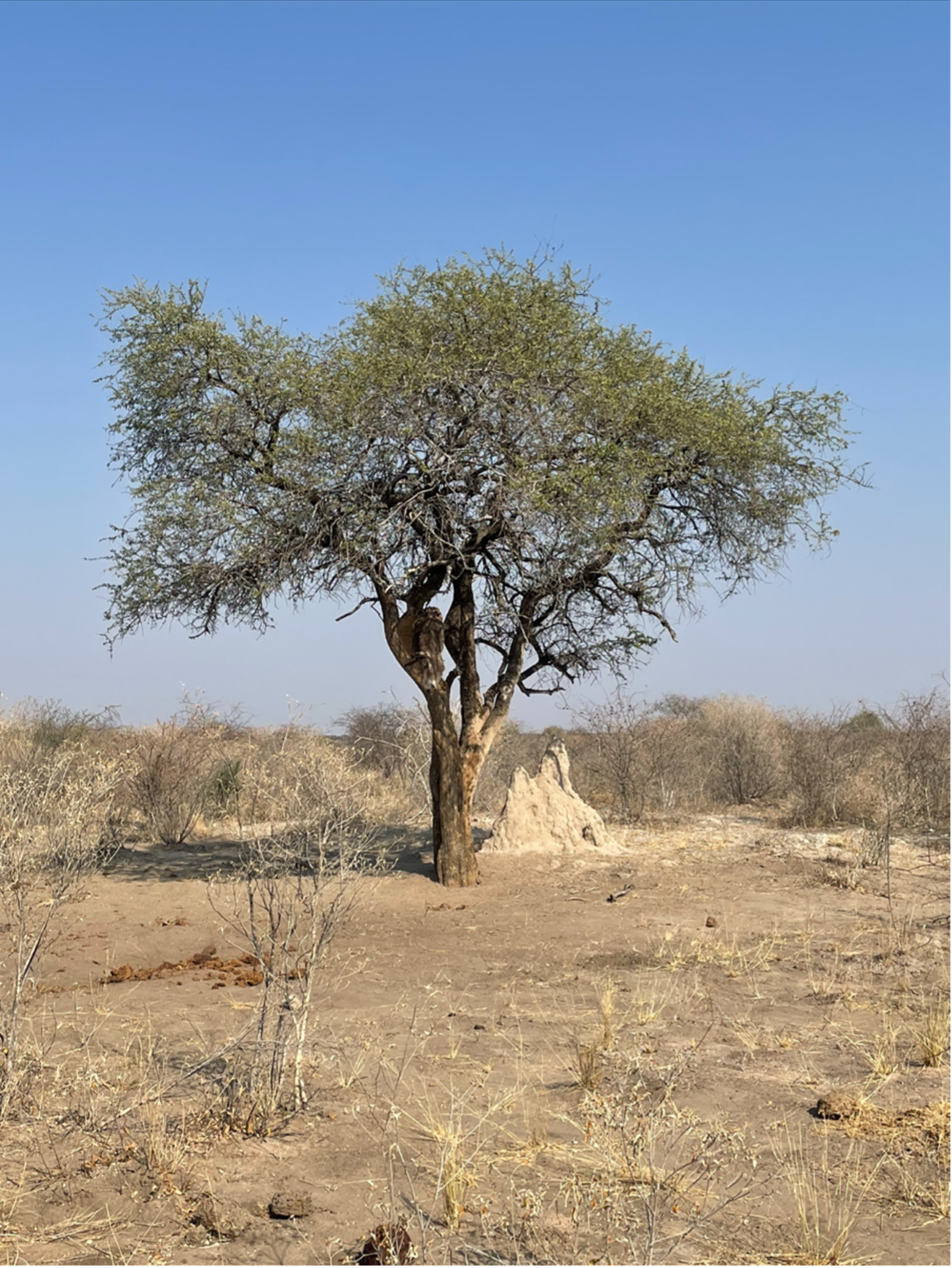
**

**Figure S1.**

Donkey carcasses used to attract large carnivores in northeastern Namibia**.** Body parts of donkeys are sometimes used to attract large carnivores for trophy hunting. Hunting of large carnivores is permitted in some areas in accordance with legal quotas and requirements (i.e., sex, age, area). This picture depicts a donkey head hung from a camelthorn tree to attract leopards in a conservancy area of northeastern Namibia. Photo taken by first author.

**Table S1.**

*Studies from the literature sample that represent various conditions and recommendations for LGDk use.*

| Study  (design; tested efficacy) | Livestock | Carnivore | Number of LGDks / Herd | Paddock (type; size) | LGDk traits (height in cm) | Regional context  (region; habitat; climate) | Implementation (bonding period; process; age) | Findings | Challenges and Limitations |
| --- | --- | --- | --- | --- | --- | --- | --- | --- | --- |
| Angst et al., 2002  (Exp; ✓) | Sheep | Dog, Lynx | 1/<200 | Small, flat pastures; <10 acres | S (≤110 cm); agile, “terrain-friendly” | Switzerland, Europe; mountainous; winter, less suitable in summer | Not specified; bonded well with the sheep | - LGDks can provide protection against lynxes under certain conditions. - Reduced predation from all five herds post-LGDk acquisition. - LGDk use also minimised damage caused by stray dogs. | - Terrain – LGDks that are large and heavy, are less effective and at increased risk of injury on steep slopes require supervision to avoid injury. - Experimental field study; minimal controls or details pertaining to LGDk traits. |
| Botha, 2018  (Obs; ✓) | Cattle, Sheep | Caracal, Jackal | 1/51 – 100  (from farmer reports) | Smaller farms; **297–3,707 acres** | M – L; 1 jenny or gelding | South Africa, Africa; hot, arid, bushveld, open plains | Raise with livestock or from weaning age. | - All three farmers who used LGDks reported reduced jackal and caracal depredation. - One reported a reduction from 91 – 100% losses to 0 – 10%. Another reported increased weaning with LGDk use. | - No details pertaining to LGDk traits or their implementation. - Observational study, minimal data sample; insufficient to assess efficacy. - Farms using LGDks were smaller than farms with other LGAs. |
| Bourne, 1994  (Rep) | Cattle, Sheep | Brown bear, Coyote, Dog, Grey wolf | 1/200 max.(est.) | Small, **well-fenced, open, level pastures** | M (111 – 137 cm); 1 jenny (and foal for large areas) or 1 jack/stallion (only if raised with herd) | Missouri, North America; not specified | 7 – 10 days in paddock adjacent to herd, followed by 7 days in a shared pen; may take several weeks; birth or weaning age rec. | - Consistent and successful LGDk use depends on individual selection, training, and realistic expectations. - LGDk reported to have chased bears. - LGDk should have some exposure to sheep or other livestock it is to protect. | - Terrain – LGDks can fall on icy or slippery areas due to their small hooves. - LGDks do not intentionally patrol the pasture looking for intruders. - Fences must be secure to avoid LGDk escaping. |
| Braithwait, 1996  (Rep) | Cattle, Sheep, Goats | Coyote, Dog | 1/40 rec. 1/200 max. | Small; **<80 acres** | M – L; 1 jenny and foal | Missouri, North America; not specified; yearlong | 4 – 6 wks; align foaling with livestock birthing period rec. | - Jennies are easier to work with, are gentle with livestock and exhibit increased aggression towards dogs. - LGDks can be conditioned to feel part of the herd by being fed at the same time and location. | - Number of LGDks – one LGDk is less effective in large pastures; however, two or more may bond with each other. - Infrastructure – solid fencing is required weak enclosures can be breached to reach nearby equines. |
| De Gabriel et al., 2022  (Exp; ✓) | Cattle | Grey wolf, Dog | 1/20 – 50 | Small to medium, **open, fenced, few trees** | L (≤145 cm); 1 stallion or gelding | Spain, Europe; not specified, study period of two years/seasons | 10 days to 6 wks, min. 1 – 2 wks rec. | - Six LGDks protected five cattle herds across four farms over 11 months. - Zero losses were recorded across all herds post-LGDk deployment, compared to 18 cattle lost in the previous 12 months (1 – 8 per herd). | - Behaviour – reports of food guarding and attacks on livestock. - Husbandry practices – low farmer engagement and lack of guideline compliance reduced LGDk efficacy. |
| Drouilly et al., 2023  (Obs; X) | Sheep | Cape fox, Caracal, Jackal | N/A | Varied terrain, **flat to rugged; 5.7 – 260 acres** | N/A | South Africa, Africa; arid, hot climate | N/A | - Jackals – 14% had used LGDks;   rating: average 1.9/5, maximum 4/5   - Caracals – 12% had used LGDks; rating: average 1.9/5, maximum 4/5 | - LGDk efficacy assessed indirectly using small sample of farmers; 12 – 14 % had used LGDks. - Observational study, no information on LGDk use (traits, number, implementation etc). - Larger farms less likely to use non-lethal methods such as LGAs. |
| Lance et al., 2023  (Rep) | Cattle, Sheep, Goats, Horses | Grey wolf | N/A | Open pastures; **0 –1,000 acres** | L (use larger breeds, e.g., Mammoth); 1 jenny | Colorado, North America; yearlong | 1 – 2 wks min. | - Provided herd protection by detecting and alerting the herd to potential threats, and using defensive tactics (chasing, kicking, etc.) to deter or drive away carnivores. | - Behaviour – can be aggressive toward dogs and require supervision during the familiarisation process. Dogs will learn to work around LGDks. |
| Landry, 2000  (Obs; ✓) | Sheep | Dog, grey wolf, Grey fox, Red fox | 1/< 50 (enclosure)  1/200 – 250 (mountain pasture) | N/A | 1 jenny (not pregnant; risk of abortion) | Switzerland, Europe; mountainous; winter, LGDk use is less suitable in summer | 1 wk; any age | - Can provide effective herd protection without special training. - Presence helped calm sheep; highly adaptable to changes in ownership, climate, and activity. - Effective against stray dogs and foxes; wolves unknown. | - Terrain – steep slopes require supervision to prevent injury. - Behaviour – aggression towards livestock and people, especially males; can pose risks to unfamiliar dogs (two fatalities reported). |
| Marker-Kraus et al., 1996  (Obs; ✓) | Cattle | Caracal, Cheetah, Jackal, Leopard | 1/<200 | Small, open pastures | M – L; 1 jenny (pregnant or with foal) | Namibia, Africa; hot, arid, bushveld, open plains | 4 – 6 wks; any age | - Report of leopard being trampled. - Successful in reducing predation from cheetahs, jackals, caracals, and dogs. - Variable reports of success. | - LGDk sex – stallions tend to break fences and become aggressive during breeding. - Husbandry – poor husbandry practices likely contributed to the reduction in LGDk efficacy. |
| Scasta et al., 2024  (Exp; ✓) | Sheep | Coyotes, Dogs | 1/50 – 114 | Varied terrain; tested in 17 – 779 acre pastures | 1 jenny | Wyoming, United States; winter | ~ 5 wks; initially kept distant from herd, then proximity decreases over 2 – 5 wks | - Provided effective flock protection and were most effective in small, flat pastures. - Herd integration periods varied with area size and social distractions. | - Context – equine neighbours can be distracting. - Terrain – large, complex pastures (>600 acres) hinder guardian effectiveness and herd integration. |
| Tischaefer, 2020  (Rep) | Cattle | Coyote | 1/300 max. | Open pastures; <600 acres | M; 1 jenny or gelding | United States, North America, not specified | 4 – 6 wks | - An LGDk should be acquired from a breeder and provided training to ensure effective use. | - N/A |
| Van Liere et al., 2013  (Obs; X) | Sheep, Goats | Grey wolf | N/A/Average herd 93 | Fenced pastures; ~ 6.7 acres | N/A | Slovenia, Europe; April to November (pasture season) | 6 – 8 wks | - Four farmers with wolf attacks and three farmers without had LGDks. - LGDk presence did not affect the number of sheep killed per attack. | - Observational study, no details pertaining to LGDk traits or implementation. |
| Walton & Field, 1989  (Obs; ✓) | Sheep, Goats | Bobcat, Coyote, Dog, Grey fox, Red fox | 1/200 max. | Open pastures; 100 – 1,000 acres (≤600 acres rec.) | M – L; 1 jenny or gelding | Texas, North America; not specified | 4 – 6 wks; weaning age rec. | - Effective against coyotes, dogs both primary carnivores implicated in livestock predation losses. - May offer some protection against foxes and bobcats. | - Terrain – decreased efficacy in large pastures, rough terrain, dense vegetation - Herd size – reduce efficacy when herds were large or scattered. - Management – LGDks should be culled or sold if they fail to perform. |

*Note.* Selected sample of studies from the literature sample relating to donkeys either as recommended (rec.) or implemented LGDk use. Studies were chosen to demonstrate the discrepancies and differences between regions, recommendations, use and reports of efficacy. Abbreviations in the first column represent the study design and were observational (Obs), report (Rep) or experimental (Exp). To indicate the efficacy of LGDks involved in a study a symbol was used to depict which studies found LGDks effective (✓) or ineffective (x).
